# Lineages to circuits: the developmental and evolutionary architecture of information channels into the central complex

**DOI:** 10.1007/s00359-023-01616-y

**Published:** 2023-03-17

**Authors:** Pratyush Kandimalla, Jaison Jiro Omoto, Elizabeth J. Hong, Volker Hartenstein

**Affiliations:** 1 Division of Biology and Biological Engineering, California Institute of Technology, Pasadena, CA USA; 2 Department of Molecular, Cell and Developmental Biology, University of California, Los Angeles, CA USA

**Keywords:** Insect brains, Central complex, Large-field neurons, Lineages, Hemibrain

## Abstract

The representation and integration of internal and external cues is crucial for any organism to execute appropriate behaviors. In insects, a highly conserved region of the brain, the central complex (CX), functions in the representation of spatial information and behavioral states, as well as the transformation of this information into desired navigational commands. How does this relatively invariant structure enable the incorporation of information from the diversity of anatomical, behavioral, and ecological niches occupied by insects? Here, we examine the input channels to the CX in the context of their development and evolution. Insect brains develop from ~ 100 neuroblasts per hemisphere that divide systematically to form “lineages” of sister neurons, that project to their target neuropils along anatomically characteristic tracts. Overlaying this developmental tract information onto the recently generated *Drosophila* “hemibrain” connectome and integrating this information with the anatomical and physiological recording of neurons in other species, we observe neuropil and lineage-specific innervation, connectivity, and activity profiles in CX input channels. We posit that the proliferative potential of neuroblasts and the lineage-based architecture of information channels enable the modification of neural networks across existing, novel, and deprecated modalities in a species-specific manner, thus forming the substrate for the evolution and diversification of insect navigational circuits.

**Supplementary Information:**

The online version contains supplementary material available at 10.1007/s00359-023-01616-y.

## Introduction

The ability to generate useful internal representations of the environment is crucial for survival. Relevant external cues must not only be detected but also integrated and mapped with respect to internal state and past experiences. In organisms that can navigate their environment, these bearings often are critical for computing appropriate motor commands. In insects, a brain region called the central complex (CX) has been shown to be involved in coordinating the complex sensorimotor transformations underlying the representation and memory of spatial information (Ofstad et al. 2011; Seelig and Jayaraman 2015; Turner-Evans et al. 2017; Green et al. 2017; Behbahani et al. 2021; Lu et al. 2022; Lyu et al. 2022), action selection (Neuser et al. 2008; Giraldo et al. 2018; Dan et al. 2021), and steering control (Martin et al. 2015; Rayshubskiy et al. 2020), as well as the integration of physiological states such as hunger (Dus et al. 2013) and sleep (Donlea et al. 2011, 2014, 2018; Liu et al. 2016).

**Fig. 1:**
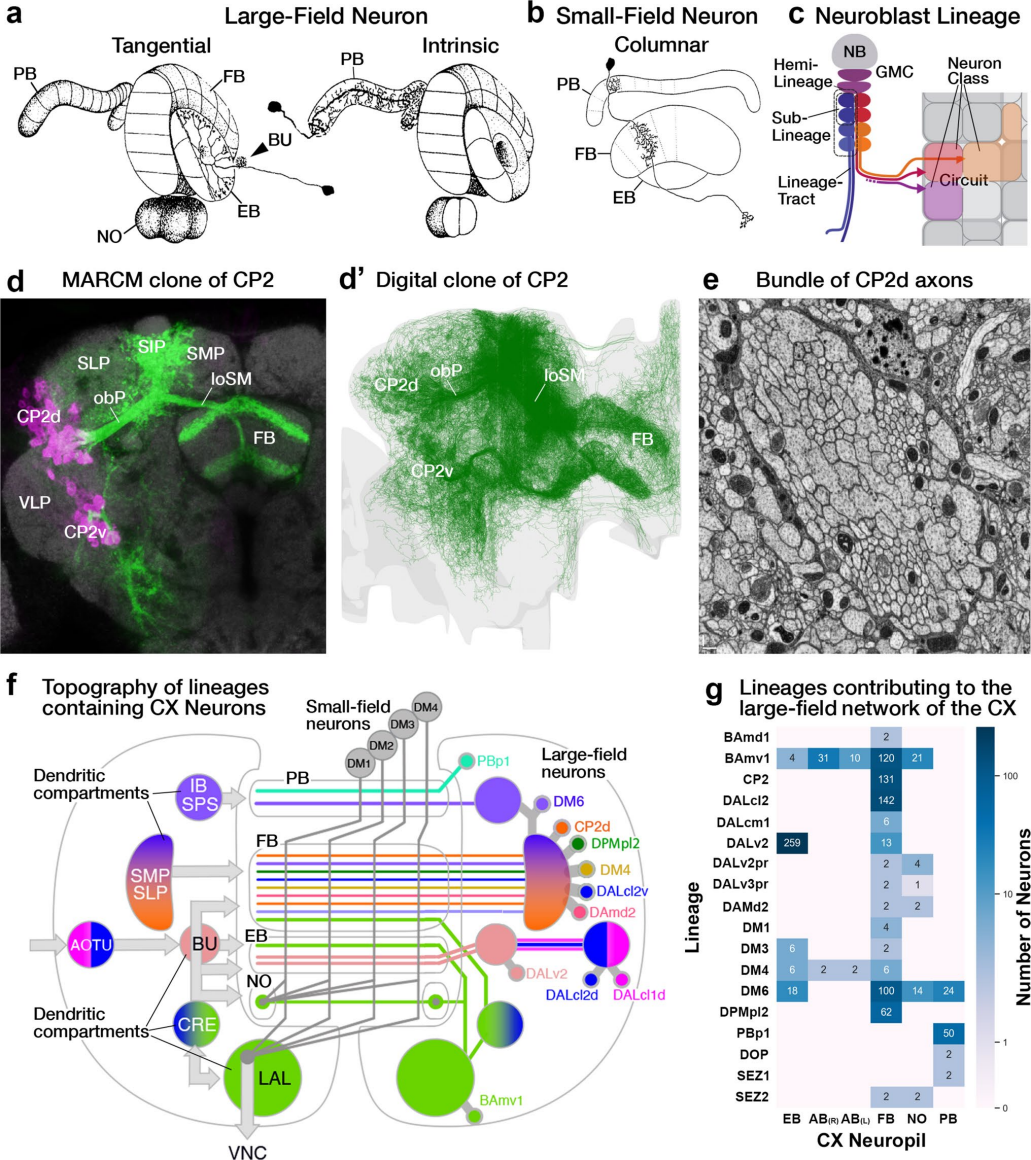
Overview of the developmental organization of the *Drosophila* central complex. **a** Schematic drawing of *Drosophila* central complex (CX; antero-lateral view) and large-field neurons. Left: DALv2 ER-neuron as a representative of tangential neuron, providing input from the bulb (BU) to the ellipsoid body (EB). Right: PBp1 (Delta7) neuron as an example of an intrinsic neuron whose arbor is restricted to a CX compartment (here: protocerebral bridge, PB). **b** Schematic drawing of CX (dorsal view) depicting representative example of small-field (columnar) neuron, with arborizations restricted to narrow volumes (glomeruli, columns) of different CX compartments. Figures adapted from (Hanesch et al. 1989).** c** Schematic of a neuronal lineage formation and projection into different neuropil compartments (grey squares). Broad neuron classes of a lineage collectively tile a few compartments, referred to as the projection envelope of the lineage, within which, individual neuron types form various circuit motifs. **d** z-projection of frontal confocal sections of *Drosophila* brain at the level of the fan-shaped body (FB). GFP-labeled MARCM clone of the CP2/DL1 lineage, consisting of a dorsal (CP2d) and ventral (CP2v) hemilineage. Neuronal cell bodies are rendered in magenta, fiber tracts and arborizations in green. Lineage-associated tracts project in characteristic patterns, as shown here for CP2d neurons that follow the oblique posterior fascicle (obP) and then the longitudinal superior medial fascicle (loSM) to reach the FB. **d’** Digital (in-silico) clone of CP2 neurons identified in the hemibrain database based on characteristic location and projection patterns. **e** Electron microscopy (EM) section of *Drosophila* brain showing CP2d axon bundle. Scale bars: 500 nm. **f** Schematic representation of CX and surrounding compartments, visualizing the topography of lineages that innervate the CX. Annotated on the left are neuropil compartments providing input to the CX: inferior bridge/superior posterior slope (IB/SPS), superior protocerebrum (SLP/SIP/SMP), anterior optic tubercle (AOTU) and bulb (BU), crepine (CRE) and lateral accessory lobe (LAL). Right half of the schematized brain shows lineages—represented by colored circles alongside their names. Position of circles roughly coincides with the location of somata clusters in the brain. Although the focus of our analysis are the large-field neurons, we also include the lineages which give rise to the small-field neurons (grey circles). Note: A novel finding from our hemibrain analysis is that the DM4 (DM1 and DM3 are hidden for brevity) lineages also give rise to a few large-field neurons. To distinguish the small- and large-field neurons of this lineage, we depict them as a separate yellow-colored circle in the right side of the schematic. Color-coded lines emanating from the different lineages interconnect the input domains of the constituent neurons with their output domains in the CX. The shading in the input domains reflects the degree of overlap (as in the SMP/SLP and CRE), or lack thereof (as in the AOTU and BU), of the arbors of the different lineages. The extension of the colored lines into the CX depicts the relative innervation patterns exhibited by these neurons. More detailed, and realistic, tract trajectories are schematized in the subsequent figures which highlight individual CX compartments. **g** Number of neurons (from both hemispheres) provided by different lineages (along vertical axis) to CX compartments (along horizontal axis). For other abbreviations see Table 1

The CX is an evolutionarily conserved structure located along the midline of the insect (and pancrustacean) brain (Strausfeld 1976; Honkanen et al. 2019). It is comprised of five distinct neuropil compartments: the protocerebral bridge (PB), upper (CBU) and lower (CBL) divisions of the central body, asymmetrical bodies (AB), and the paired noduli (NO) (Fig. 1a) (Hanesch et al. 1989; Ito et al. 2014; Wolff and Rubin 2018). In *Drosophila*, the CBU and CBL are referred to as the fan-shaped body (FB) and ellipsoid body (EB), respectively. The neuronal constituents and synaptic interactions within and across these compartments organize the CX network in a grid-like fashion, which results in a distribution of synaptic and neuronal adhesion markers in a regular pattern of layers and columns. These patterned elements serve as anatomical landmarks in the CX (Hanesch et al. 1989; Wolff et al. 2015; Wolff and Rubin 2018; Omoto et al. 2018). The columnar elements of this grid referred to as small-field neurons, interconnect the CX neuropils in the antero-posterior axis. They have been shown to perform the signal transformations required to interpret spatial information (Fig. 1b) (Seelig and Jayaraman 2015; Turner-Evans et al. 2017, 2020; Green et al. 2017; Lu et al. 2022; Lyu et al. 2022). Perpendicular to the columnar elements are the large-field or tangential neurons, which primarily interconnect the CX neuropils with lateral compartments and form the majority of the input network to the CX (Fig. 1a) (Heinze and Reppert 2011; Homberg et al. 2011; el Jundi et al. 2014, 2015; Omoto et al. 2017; Donlea et al. 2018; Okubo et al. 2020; Currier et al. 2020; Hardcastle et al. 2021; Matheson et al. 2022). Despite the vast anatomical, behavioral, and ecological diversity across insects, detailed morphological and functional analyses have revealed striking homologies among the small- and large-field neurons of the CX across species (Heinze and Homberg 2007; Heinze and Reppert 2011; Homberg et al. 2011; Omoto et al. 2017; Honkanen et al. 2019; Pisokas et al. 2020; Hardcastle et al. 2021; Sayre et al. 2021). This remarkable conservation highlights the complementary phylogenetic and ontogenetic circuit assembly mechanisms that maintain stereotypy in this structure while retaining the flexibility for functional diversification driven by selective pressure.

Insect brains develop from ~ 100 pairs of embryonic stem cells, called neuroblasts, that proliferate in a highly stereotypical manner to give rise to uniquely identifiable “lineages” of sister neurons (Malzacher 1968; Richards et al. 1976; Zacharias et al. 1993; Broadus and Doe 1995; Younossi-Hartenstein et al. 1996; Truman and Ball 1998; Urbach and Technau 2003). Neuroblasts divide asymmetrically to generate one daughter cell that is large and remains in contact with the overlying ectoderm and another that is small and comes to lie at the basal surface of its larger sibling (Fig. 1c). The large daughter cell maintains the proliferative fate of the mother neuroblast (“self-renewal”), while the small daughter cell (“ganglion mother cell” or GMC) undergoes one more molecularly asymmetric division. This division produces two neurons (“A” and “B”) differing in Notch signaling activity (Truman et al. 2010). The series of “A” and “B” neurons sequentially produced from the GMCs form their own “A” hemilineage and “B” hemilineage, respectively. Individual hemilineages form characteristic tracts (Fig. 1c, e), surrounded by glia, as the constituent neurons enter, traverse, and interconnect the neuropil volume (Dumstrei et al. 2003; Spindler and Hartenstein 2011; Lee et al. 2020). Many lineages lose an entire hemilineage via apoptotic cell death and as a result possess a single tract (Kumar et al. 2009a, b). Lineages in which both hemilineages survive have two tracts (Lovick et al. 2013, 2016). A small group (eight in all) of atypical neuroblasts, divide asymmetrically to give rise to a series of “intermediate neural progenitors” (INP), each of which produces its own small “INP lineage” in a neuroblast like fashion. These neuroblast lineages are referred to as type II lineages, and tend to generate a larger number of neurons and extend more tracts than the “normal” type I lineages. Often visualized by immunohistochemical labeling of glial, cell adhesion, or cytoskeletal markers, the organization of lineage tracts are similar across species and are useful guides to compare the morphology and development of insect brains (Bressan et al. 2015; Farnworth et al. 2020, 2022). The sequential gene expression profiles of each neuroblast shape the properties of the neurons born during specific temporal windows. These groups of neurons, referred to as “sublineages”, represent individual neuron types and form the basic modules of circuits (Fig. 1c) (Harris et al. 2015; Hartenstein et al. 2015; Lovick et al. 2017; Sullivan et al. 2019; Mark et al. 2021). Experimental manipulation of the duration of neuroblast division windows and/or the gene expression profiles within these windows have been shown to produce numerical, morphological, and functional aberrations in targeted sublineages—phenomena that underlie the modification of neuronal circuits over the course of speciation (Truman and Ball 1998; Sullivan et al. 2019; Farnworth et al. 2022). Thus, from a structural, functional, developmental, and evolutionary perspective, lineages, hemilineages, and sublineages are the key to understanding insect nervous systems.

In *Drosophila melanogaster*, access to a plethora of genetic tools has enabled finer dissection of the developmental and anatomical properties of lineages. Clonal labeling and manipulation of the derivatives of individual neuroblasts, has proven invaluable for visualizing their neuropil/compartment innervation, gross connectivity patterns, characterizing the molecular mechanisms that define individual sublineages (neuron types), as well as determining their birth order (Jefferis et al. 2001; Komiyama et al. 2003; Ito et al. 2013; Yu et al. 2013; Lovick et al. 2013; Wong et al. 2013; Sen et al. 2014; Omoto et al. 2018; Andrade et al. 2019; Sullivan et al. 2019; Lee et al. 2020). Together with recordings of neural activity in targeted sublineages, these developmental analyses are a powerful source of insight into information organization and processing in the fly nervous system (Omoto et al. 2017; Hardcastle et al. 2021). Detailed insight into the connectivity of the constituent circuit elements have become possible with recent advancements in large-scale electron-microscopy (EM) based image acquisition and processing techniques (Saalfeld et al. 2009, 2010; Eichler et al. 2017; Zheng et al. 2018; Li et al. 2019, 2020; Lu et al. 2019; Scheffer et al. 2020; Turner-Evans et al. 2020; Sheridan et al. 2021; Phelps et al. 2021; Hulse et al. 2021). Commonly referred to as “connectomes”, these EM datasets provide an unparalleled synaptic-resolution view of circuit architectures, revealing their organizational and numerical complexity (Zheng et al. 2018; Scheffer et al. 2020; Li et al. 2020; Hulse et al. 2021). Additionally, connectomes also contain a significant amount of structural information, including features that could be leveraged as proxies for the developmental trajectory of the nervous system. Integrating long-time-scale developmental data with synaptic data from connectomes would provide novel frameworks to understand circuit motifs that shape complex neural computations.

Here we systematically assign neuroblast lineage information to the “hemibrain” connectome (Scheffer et al. 2020) and examine the architecture of the large-field network of the CX (Fig. 1f, g). We find that synaptic output from neurons derived from specific lineages are spatially organized within each CX compartment, and we describe the structure of their inputs from lateral neuropils. An important aspect of this work, beyond elucidating the developmental organization of the CX input channels, is to facilitate comparative analyses which may yield general insight into the evolution of insect nervous systems. As an entry point towards this goal, we compare morphological renderings of large-field neurons in other insect species and propose developmental parallels across circuit motifs. Finally, we attempt to reconstruct the core organizational and assembly principles that have facilitated the extensive cooption, deprecation, and processing of novel information streams, a bedrock for rapid diversification, of the central complex.

## Materials and methods

### Dataset

We analyzed the hemibrain connectome, the details of which have been described by Scheffer et al. (2020).

### Software and code availability

We accessed the hemibrain version 1.2.1 (publicly available at https://neuprint.janelia.org) using the python library, NAVis (https://github.com/navis-org/navis). Additionally, we wrote custom cypher queries to mine the dataset via the neuPrint + browser interface. Raw EM images were accessed through the neuroglancer (https://github.com/google/neuroglancer) viewer on neuPrint + (Scheffer et al. 2020; Plaza et al. 2022). All subsequent analysis was performed in python.

Analysis code will be made available at https://github.com/KandimallaPrat/Lineages2Circuits upon publication.

### Neuron tract generation

Tracts were generated using neuron skeletons imported from hemibrain through NAVis. First, each neuron was “cleaned” to remove branching artifacts that remain after the skeletonization of 3D volumes. This involved isolating branches smaller than a set threshold (15 nodes, i.e. ~ 120 nm) and discarding them if they were not associated with a connector (T-bar or post-synaptic density). Next, neurons were cut at each branch point and large fragments (fragments > 2500 nm) isolated. Fragments that were smaller than this threshold were subject to secondary scrutiny to determine their fate. If these smaller fragments were greater than 80 nm and the number of connectors associated with the fragment were less than 1% of the numerical value of the cable length, they were retained as part of the tract. Of the selected fragments, any that were less than 2.5 times the length of the largest fragment were also discarded. Occasionally, these selection criteria incorporated fragments at the terminals of the neuron skeleton. To automate their exclusion, any fragments that were more than 5000 nm away from either ends of the largest fragment were also discarded.

### EM bundle identification

Using previously described anatomical landmarks for each of the tracts and fascicles, we identified sections in the EM volume that depict these bundles (Lovick et al. 2013; Wong et al. 2013). Individual bundles were distinguished based on the electron-dense glial staining around clusters of parallel neuronal fibers.

### MARCM lineage clone generation

We utilized mosaic analysis with a repressible cell marker (MARCM) to stochastically label individual neuroblast lineages in the adult brain with GFP (Lee and Luo 1999). Mitotic recombination was achieved by temperature shifting late first/early second instar larvae (~ 12–44 h after larval hatching) to 38 °C for 30 min-1 h. We used animals bearing the following genotypes:

1. hs-flp/ + ; FRTG13, UAS-mCD8::GFP/FRTG13, tub-GAL80; tub-Gal4/ + 

OR

(2) FRT19A, tub-GAL80, hs-flp; UAS-mCD8::GFP/elavC155-Gal4, FRT19A; UAS-CD8::GFP/ + 

Lineages that contribute large-field elements to the central complex were subsequently analyzed; cellular compartments of lineages were pseudo-colored (cell body clusters, primary axon tracts, projection envelope of neurites) and compared to the in-silico clone from the hemibrain connectome.

### Immunohistochemistry

Adult brains were dissected and stained as previously described in Omoto et al. (2018).

The following antibodies were provided by the Developmental Studies Hybridoma Bank (Iowa City, IA, United States): rat anti-DN-cadherin (DN-EX #8, 1:20), mouse anti-neuroglian (BP104, 1:30). Chicken anti-GFP (Abcam #ab13970, 1:1000) was also used. Secondary antibodies, IgG1 (Jackson ImmunoResearch; Molecular Probes) were used at the following dilutions: Cy5- conjugated anti-mouse (1:300), Cy3-conjugated anti-rat (1:300). We used Alexa 488-conjugated anti-chicken (1:1000) from Thermo Fisher Scientific.

### Confocal microscopy

Samples were mounted along the antero-posterior (A-P). For a clearer view of the posterior lineages, samples were also mounted along the postero-anterior (P-A) orientation. Whole-mounted brains were imaged using confocal microscopy [LSM 700 Imager M2 using Zen 2009 (Carl Zeiss Inc.)]. Series of optical sections were imaged using a 40 × oil lens with a numerical aperture of 1.3, a zoom factor of 1.0, at 1.2-µM intervals, and 1024 pixel × 1024 pixel resolution. Digitized images of confocal sections were processed in FIJI (Schindelin et al. 2012).

### Comparative species analysis

Our comparative analysis used a two-pronged approach. We conducted a literature survey to identify neurons that have been identified and described in other (not *Drosophila melanogaster*) species. We used their rendering of neurons as well as the authors’ descriptions to assign these neurons to tracts/lineages. Where available and registered into the insect brain database (Heinze et al. 2021), we also used the image stacks, 3D renderings, or topology maps of these neurons to ascertain our classification.

### Visualizations

All schematics and visualization aids were prepared using Inkscape, Adobe Illustrator, and Photoshop.

## Results

### Clonal origins of the hemibrain: in-vivo and in-silico lineages and tracts

As it undergoes asymmetric division, a neuroblast generates a lineage of daughter neurons, that remain in close proximity and extend filopodia/growth cones along well-defined trajectories, often encased by a thickened glial sheath, towards the neuropil. This mode of development ascribes three characteristic features to each lineage, all of which can be visualized through clonally labeling (activation of a GFP marker in the neuroblast) the neurons of that lineage: (1) location of the somata in the cortex (rind), (2) location of entry of the lineage tract into the neuropil and the following trajectory, and (3) the projection envelope of the constituent neurons. Using these criteria, previous studies have identified ~ 100 lineages and their associated tracts per hemisphere and traced their development through the larval and pupal stages (Fig. 1d; Supp. Figure 1) (Pereanu et al. 2010; Lovick et al. 2013; Wong et al. 2013). These descriptions provide a valuable starting point for annotating lineages in the hemibrain.

In the raw EM volume of the hemibrain, as well as a second, similar dataset of the Female Adult Fly Brain (Zheng et al. 2018), lineage-associated tracts are easily distinguishable near their location of entry into the neuropil by the large number of parallel neuronal profiles surrounded by electron-dense glia (Fig. 1e). Using previously described anatomical landmarks for each of the tracts and fascicles, we identified sections in the EM volume which depict these bundles (Lovick et al. 2013). Tracts typically contain between 50 and 150 fibers. When visualizing the full skeletons of all neurons that form part of a given tract, we see a “digital clone” (“in-silico” clone), which not surprisingly, closely resembles the “genetic clone” obtained through neuroblast labeling (Fig. 1d, e; Supp. Figure 1). To aid the clonal analysis, we also generated a map of lineage tracts in the hemibrain by isolating the backbones of neuron skeletons (see Methods; Supp. Figure 1). The position and trajectory of the tracts as well as the position within the annotated EM bundles enabled us to assign most neurons in the hemibrain to an identified lineage—with either feature largely resolving ambiguities in the other. For lineages with known entry points outside the hemibrain volume, such as in the subesophageal zone, we used the projection envelopes of the neurons and their tract trajectories to best assign identities (Table 1).

**Table 1:**
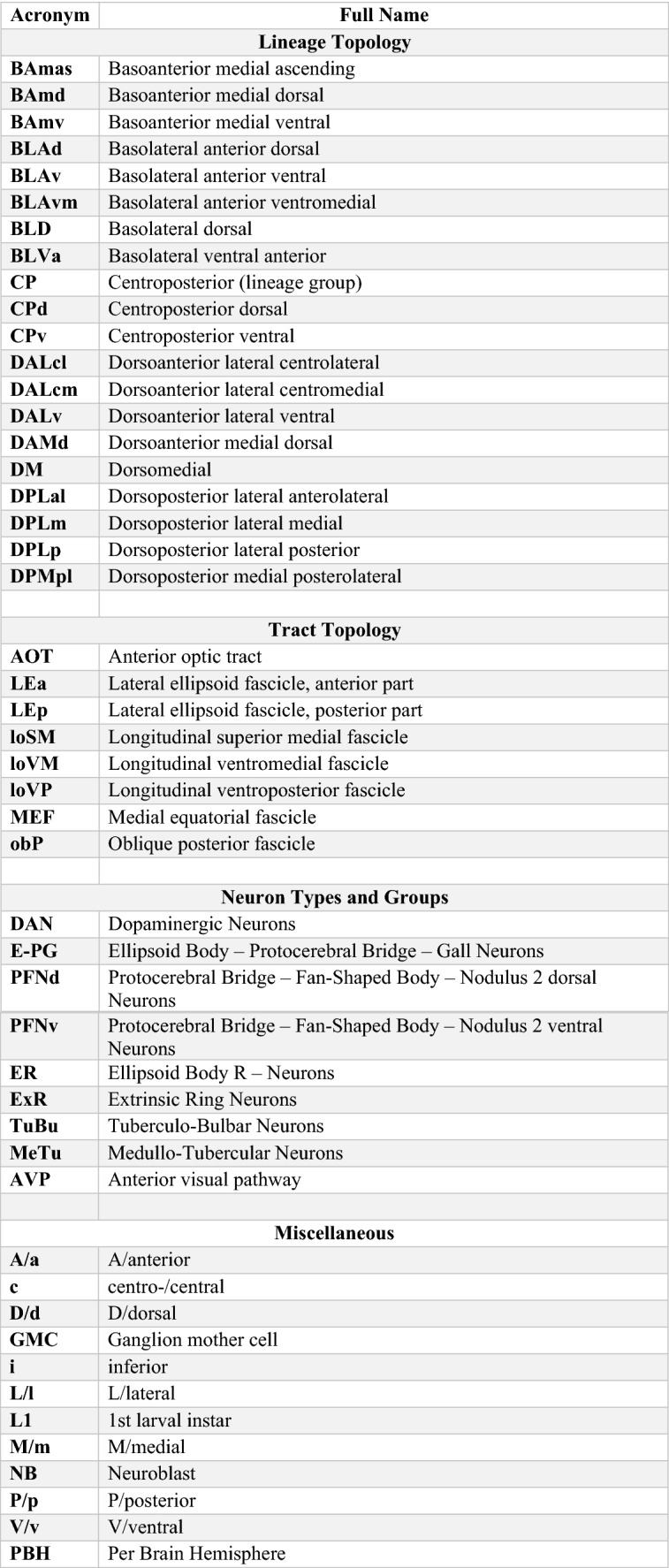
Acronyms and abbreviations used

Note that for the adult *Drosophila* brain, two different terms (e.g., “BAmv1” and “LALv1”) exist for many lineages (Table 2). The first term is part of the nomenclature system introduced for brain lineages on the basis of their characteristic axon tracts, which remain detectable throughout development (Pereanu and Hartenstein 2006; Cardona et al. 2009; Lovick et al. 2013; Hartenstein et al. 2015). The second system of lineage terms was introduced by Ito et al. (2013) and Yu et al. (2013) for lineages (“clonal units”) as visualized by clonal analysis in the adult brain. We use both names when introducing a lineage in the text for the first time; subsequently, for reasons of brevity and continuity with our tract-based analysis, we will use the tract-based term (see Table 2 for nomenclature). We make an exception for the “dorsomedial” lineages (DM1-6), for which a nomenclature was introduced previously in a set of studies that investigated the unorthodox proliferation pattern of these special (“type II”) lineages (Bello et al. 2008; Boone and Doe 2008; Izergina et al. 2009).

**Table 2:**
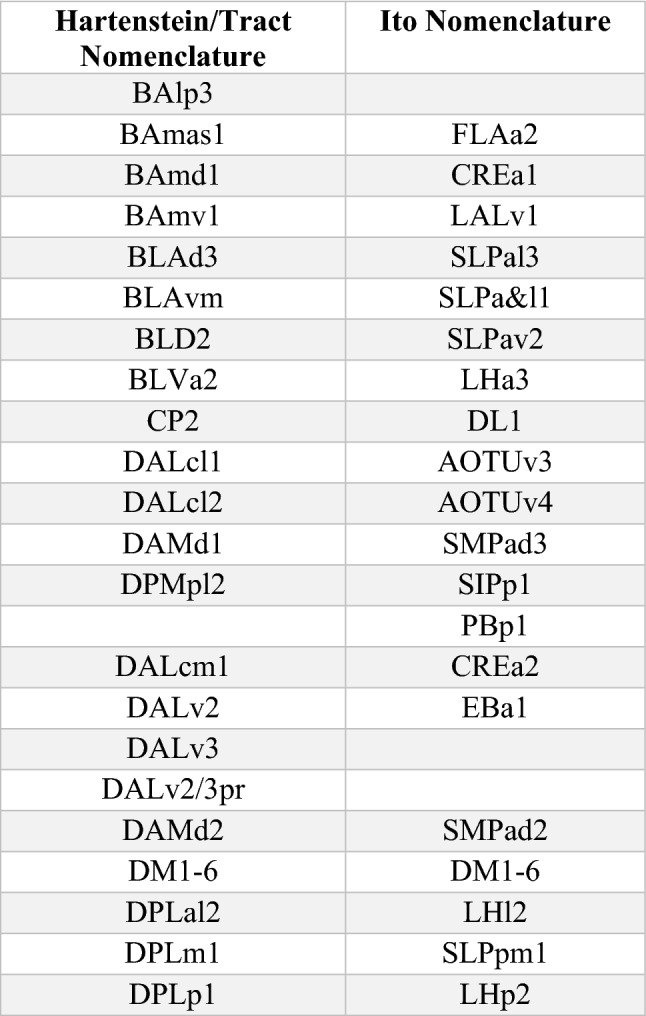
Lineage nomenclature

In the present study, we focused our analysis on the lineages that give rise to neurons of the CX. Consistent with previous studies (Yu et al. 2013; Yang et al. 2013; Wong et al. 2013), we found four lineages (DPMm1/DM1, DPMpm1/DM2, DPMpm2/DM3, and CM4/DM4) that constitute the small-field/columnar neurons of the CX. We also identified fourteen lineages (which includes DM1, DM3, and DM4—not previously thought to do so) that generate the large-field neurons in addition to other non-CX neurons (Fig. 1f).

The CX large-field lineages display a lot of diversity in their numerical and spatial innervation patterns across the CX, ranging from containing a single neuron per hemisphere restricted to a single neuropil to forming over a hundred spanning multiple compartments (Fig. 1g; Supp. Figure 1; Supp. Table 1). That being said, we identified seven “major” lineages, DALv2/EBa1, DALcl2/AOTUv4, DPMpl2/SIPp1, BAmv1/LALv1, CM3/DM6, CP2/DL1, and PBp1, which devote a large fraction of their respective neurons to the CX (Fig. 1g; Supp. Table 1). The remainder (BAmd1/CREa1, DALcm1/CREa2, DAMd2/SMPad2, DM1, DM3, DM4, DALv3, two unidentified SEZ lineages) each have about 1–5 neurons targeting the CX. Additionally, we also found 6 (3 per brain hemisphere i.e. PBH) neuromodulatory large-field neurons with their somata and tracts outside the hemibrain volume. While we (and Scheffer et al. (2020)) were able to match them with neurons described using genetic approaches, their deviation from major bundles and absence in any observed lineage clone precluded us from assigning a developmental identity to them (Busch et al. 2009; Yu et al. 2013; Wong et al. 2013; Lin et al. 2014; Wolff et al. 2015; Hartenstein et al. 2017). These neurons are likely embryonically born (primary) and transdifferentiate during pupation to be incorporated into the CX of the adult brain.

DALv2 gives rise to the largest number of CX-projecting large-field neurons (270 across both hemispheres), which almost exclusively project to the EB (ER-neurons; Fig. 1g). Within the DALv cluster, we also identified the bona fide primary tracts of DALv2 (DALv2pr) and DALv3 (DALv3pr; Hartenstein and Kandimalla, in preparation). The projection envelopes of these embryonically born CX components of DALv2/3 fall within the FB and NO. BAmv1 and DM6 are the broadest lineages, with their constituent neurons collectively innervating the entirety of the CX. BAmv1 is the sole contributor to the AB and a major contributor to the FB and NO, in addition to forming 4 (2 PBH) dense large-field neurons of the EB. DM6 forms neurons spanning the PB (PB_G2-9_.b-IB.s.SPS.s from Wolff et al. (2015)), EB, and FB. A subset of dopaminergic DM6 FB neurons also extends projections into the NO—a morphological feature shared by the two (1 PBH) CX neurons of DAMd2. Complementing DM6, the lineage PBp1 gives rise to the more typical large-field neurons of the PB. However, unlike the other lineages, the projection envelope of PBp1 is entirely contained within the PB, with only a single pair of neurons per hemisphere having sparse dendritic arbors in the superior posterior slope (SPS) (Yu et al. 2013; Wolff et al. 2015). The remainder of the lineages (BAmd1, CP2, DALcl2, DALcm1, and DPMpl2) contain large-field neurons that selectively innervate the FB, making it the most neuron-rich and developmentally diverse neuropil of the CX (see supplementary files for 3D interactive plots of CX large-field neurons from each lineage). Overall, each central complex compartment is assembled by a unique assortment of developmental units—highlighting the ontogenetic complexity of this brain region.

What structural and functional properties does the lineage composition confer each compartment? In order to address this question, we examined the connectivity and projection biases across the large-field neurons and their upstream partners in lateral neuropils. We started with the EB, the compartment that has historically received the most developmental (Omoto et al. 2017; Lovick et al. 2017) and functional (Seelig and Jayaraman 2013, 2015; Omoto et al. 2017; Sun et al. 2017; Shiozaki and Kazama 2017; Donlea et al. 2018; Giraldo et al. 2018; Warren et al. 2019; Hardcastle et al. 2021) attention in *Drosophila*, in addition to being widely studied in other species (Heinze and Reppert 2011; Homberg et al. 2011; el Jundi et al. 2014, 2015).

### Assembling the annuli: architecture of the ellipsoid body

The large-field neurons of the ellipsoid body (EB) display characteristic circular arbors, earning them the moniker “ring neurons” (Hanesch et al. 1989). This term represents two major large-field neuron types, “R-neurons” and “Extrinsic ring neurons” (ExR) (Hanesch et al. 1989; Omoto et al. 2018). Often used as an abbreviation for “ring neuron”, the term R-neuron exclusively refers to the DALv2-derived EB population (Hanesch et al. 1989; Omoto et al. 2018). Currently, renamed to “ER-neurons”, to avoid conflicts with photoreceptor nomenclature (Hulse et al. 2021), most of these neurons form the terminal leg of the anterior visual pathway (AVP)—as discussed in detail in the first part of the following section (Omoto et al. 2017, 2018; Hardcastle et al. 2021). The ExR neurons, on the other hand, are collectively derived from four other lineages—the anteriorly located BAmv1 as well as the posteriorly located DM6, DM4, and DM3. They typically display large arborization outside the EB with varying polarity across the neuropils (Omoto et al. 2018; Hulse et al. 2021). In the second part of the following section, we will examine the ER-neurons that are not part of the AVP as well as the ExR neurons uncovered in the hemibrain dataset.

#### Visual inputs to the central complex by developmentally and functionally distinct neuronal populations

**Fig. 2:**
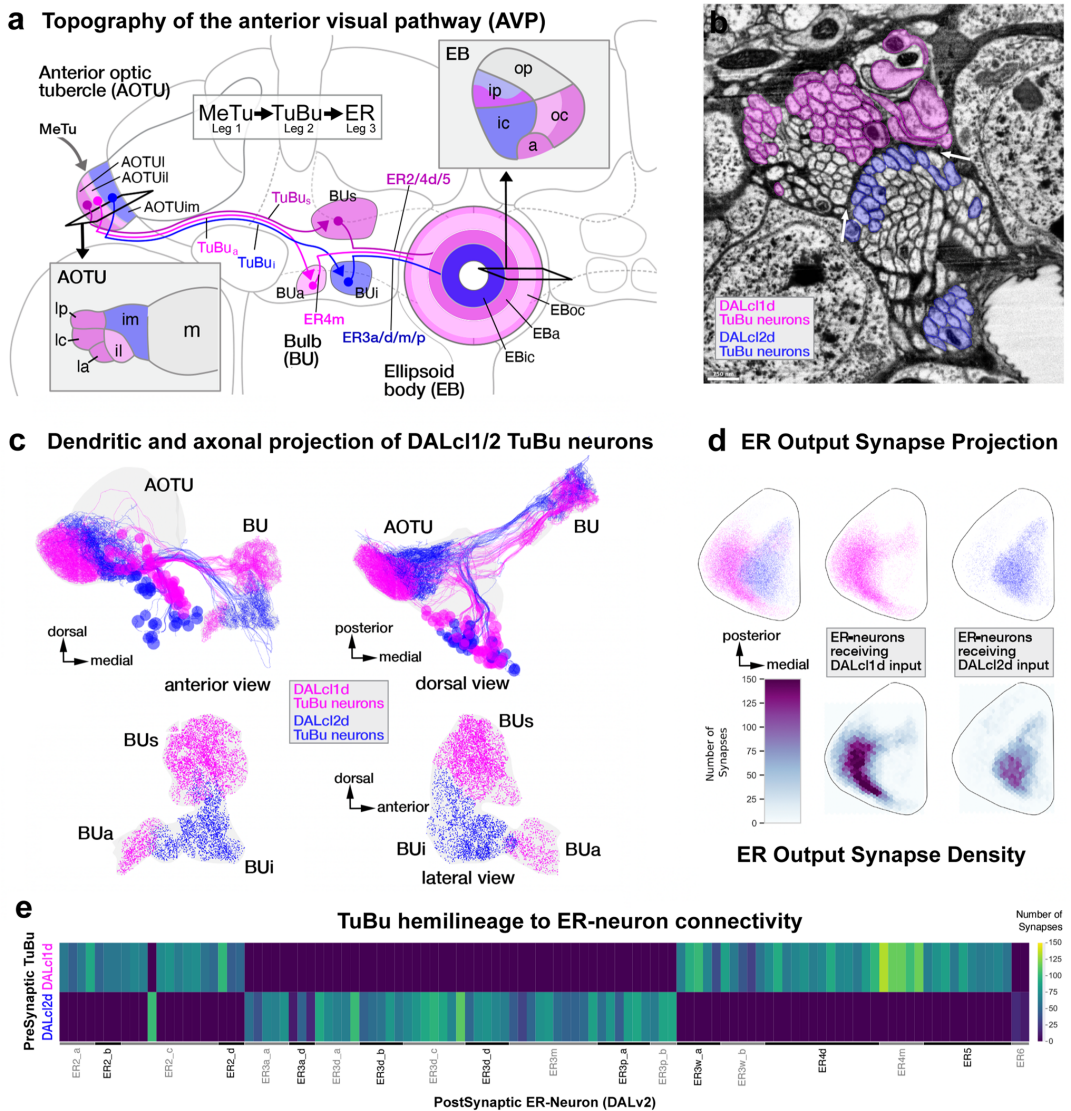
Anterior visual pathway provides developmentally segregated visual input to the ellipsoid body (EB) via anterior optic tubercle (AOTU) and bulb (BU). **a** Schematic frontal section of the brain hemisphere at the level of AOTU and EB, visualizing neuropil compartments and lineages forming the AVP. Two hemilineages, DALcl1d (magenta) and DALcl2d (blue) form the tuberculo-bulbar neurons that connect the lateral and intermediate domains of the AOTU to the BU. The TuBu_s_ neurons (dark magenta) innervate the lateral anterior, lateral intermediate, and lateral posterior AOTU (AOTUla, AOTUli, AOTUlp) and project to the superior BU (BUs); TuBu_a_ neurons, also derived from DALcl1d, link the intermediate lateral AOTU (AOTUil) to the anterior bulb (BUa). TuBu_i_ neurons, descending from DALcl2d, project from the intermediate medial AOTU (AOTUim) to the inferior bulb (BUi; inset at the left shows AOTU compartments at higher magnification). These three separate channels continue towards the EB. Discrete sublineages of DALv2 generate the outer ring neurons (ER2, ER4d, ER5, ER3w; magenta) that connect the BUs with the anterior and outer central domain of the EB (EBa, EBoc); ER4m (light magenta) projects from BUa to the EBoc; inner ring neurons (ER3a/d/m/p; blue) link the BUi to the inner central and inner posterior EB (EBic, EBip; inset at the right shows EB domains in a horizontal section of the left half of the EB). The MeTu (optic-lobe derived), TuBu (DALcl1/2d), and ER-neurons (DALv2) constitute the first, second, and third legs of the AVP respectively. **b** EM section of DALcl1d/DALcl2d axon tracts. TuBu neurons are shaded in magenta (TuBu_s_ and TuBu_a_) and blue (TuBu_i_). Non-colored axons of DALcl1/2d project to targets other than the BU. **c** Plot of TuBu neurons (top) rendered in magenta (DALcl1d-derived) and blue (DALcl2d-derived); anterior view (left) and dorsal view (right). Plot of TuBu output synapses in the bulb is shown at the bottom. **d** Circular projection of the EB cross sections along the antero-radial axis (outline); with AVP ER-neuron output synapses similarly collapsed onto a single plane. Synapses (dots) are color-coded by upstream TuBu lineage (downstream of DALcl1d; magenta) (downstream of DALcl2d, blue). Each dot represents a T-bar and is shaded by relative input strength from the TuBu neurons (normalized to the strongest TuBu to ER connection). Plots at the bottom show synapse density for the two groups of ER-neurons. **e** Heatmap showing synapse numbers of DALcl1d-derived TuBu (top) and DALcl2d-derived TuBu neurons on different subclasses of ER-neurons (horizontal axis)

The *Drosophila* AVP is a three-legged pathway that transmits multimodal visual information from the optic lobe to the CX (Omoto et al. 2017; Sun et al. 2017; Shiozaki and Kazama 2017). The first leg of this pathway is formed by the optic lobe-derived medullo-tubercular (MeTu) neurons, which receive inputs in the medulla (ME) and project to the lower unit of the anterior optic tubercle (AOTU) via the anterior optic tract. In the AOTU, MeTu neurons primarily provide inputs to the tuberculo-bulbar (TuBu) neurons, forming the second leg of the AVP. TuBu neurons have been shown to be derived from two lineages, specifically the dorsal hemilineages of DALcl1 and DALcl2 (DALcl1d and DALcl2d), and interconnect the AOTU to the tripartite bulb (BU; Supp. File 1) (Omoto et al. 2017; Lovick et al. 2017). In the BU, this pathway culminates with the TuBu neuron synapsing onto the third leg of the pathway, the DALv2-derived ER-neurons (Fig. 2a) (Omoto et al. 2017, 2018; Hardcastle et al. 2021). Here we encounter the (exceptional) case where not only the large-field neurons themselves but also their upstream partners are strictly defined by their lineage association. Visual processing across all legs of the AVP is segregated into three topographically ordered parallel streams, which collectively encode polarized (Hardcastle et al. 2021) as well as small and broad bright unpolarized light stimuli (Seelig and Jayaraman 2013; Omoto et al. 2017; Sun et al. 2017; Shiozaki and Kazama 2017).

Due to the disparate developmental origins as well as the absence of their primary dendritic domains (located in the ME) in the hemibrain volume, we chose to exclude the MeTu neurons from our analysis. However, we still see spatial tiling of the axonal tufts of individual MeTu neurons in the AOTU, which is concomitant with previous anatomical and functional data (Omoto et al. 2017; Timaeus et al. 2020; Hardcastle et al. 2021). Their downstream partners, the TuBu neurons, are entirely within the imaged volume. This allowed us to characterize their morphological and synaptic profiles in relation to their developmental origins. TuBu neurons project in two distinct adjacent bundles which we annotated as the dorsal hemilineages of DALcl1 and DALcl2 (Fig. 2b; Supp. Table 2). The somata of TuBu neurons, located in the anterior cortex flanking the AOTU, extend projections posteriorly along the ventral surface of the AOTU before branching at its postero-ventral face. The proximal (dendritic) branch of each neuron projects laterally and arborizes in a spatially restricted manner in the lower unit of the AOTU (Fig. 2c top).

The lower unit of the AOTU can be divided into three domains along the medial–lateral axis (intermediate-medial, intermediate-lateral, and lateral) based on the expression pattern of the neuronal adhesion molecule, N-cadherin (N-cad; Fig. 2a) (Omoto et al. 2017). Most TuBu neurons selectively arborize in one of these domains, where they also maintain a dorso-ventral topography (Omoto et al. 2017; Timaeus et al. 2020; Hardcastle et al. 2021; Hulse et al. 2021). This dual axis of the organization not only segregates the type of MeTu input (lateral-medial axis) and thus the visual modality inherited by the different TuBu neurons, but also preserves the retinotopy of these inputs from the ME (dorso-ventral axis) (Omoto et al. 2017; Hardcastle et al. 2021). Corroborating these anatomical data, we see spatially segregated innervation patterns of every TuBu type in the hemibrain, which we can assign to the appropriate N-cad domains (Fig. 2c top; Supp. File 1). Furthermore, we confirm the complementary innervation of DALcl1d and DALcl2d TuBu neurons in the AOTU, with the former occupying the intermediate-lateral (AOTUil) and lateral domains (AOTUl), and the latter being restricted to the intermediate medial domain (AOTUim; Fig. 2c; Supp. File 1) (Omoto et al. 2017).

The distal (axonal) branch of every TuBu neuron extends medially, traversing dorsally of the peduncle and posterior to the vertical lobe of the mushroom body (MB), before continuing ventrally towards the BU (Fig. 2c top). Each neuron targets one of three anatomical partitions of the BU—superior, inferior, and anterior—and arborizes in a glomerular fashion (Supp. File 1; Fig. 2c). As in the AOTU, a clear anatomical segregation between the DALcl1d and DALcl2d TuBu neurons is visible in the BU (Fig. 2c bottom) (Lovick et al. 2017; Omoto et al. 2017). DALcl1d TuBu neurons innervate the anterior (BUa) and superior (BUs) partitions, leaving the DALcl2d TuBu neurons to occupy the inferior partition (BUi; Fig. 2c, bottom; Supp. File 1). This distinction is even more evident in the synapse density map of the BU, displaying a sharp split between the BUs (DALcl1d; magenta) and BUi (DALcl2d; blue; Fig. 2c bottom right)—a boundary that is difficult to draw at the light-microscopy level using antibodies such as N-cad and nc82 (Brp) (Omoto et al. 2017, 2018; Hardcastle et al. 2021).

Complementing our anatomical characterization, functional recordings of TuBu neuron terminals have also revealed compartmentalization of response properties in the AOTU and the BU (Omoto et al. 2017; Sun et al. 2017; Shiozaki and Kazama 2017; Hardcastle et al. 2021). Targeted expression of calcium indicators in neurons projecting from the AOTUl to the BUs, the DALcl1d TuBu neurons collectively referred to as TuBu_s_ (Supp. Table 2), reveal that they are excited in response to bright objects in small retinotopically organized visual fields in the ipsilateral hemisphere. This retinotopy is preserved in the spatial positioning of the tufts and glomeruli in the AOTU and BU, respectively (Omoto et al. 2017; Sun et al. 2017; Shiozaki and Kazama 2017). Located adjacently, the AOTUil to BUa projecting TuBu neurons, TuBu_a_ (TuBu01 in the hemibrain), represent a small and highly specialized DALcl1d population which encodes the angle of polarization of light incident on the dorsal rim area of the eyes (Hardcastle et al. 2021). Finally, the AOTUim to BUi projecting neurons, the DALcl2d-derived TuBu neurons (collectively TuBu_i_; Supp. Table 2), have very broad overlapping receptive fields located in both the ipsi- and contralateral hemispheres. TuBu_i_ neurons are excited by bright objects entering the contralateral visual hemifield and inhibited by the same in the ipsilateral visual hemifield. They also display a secondary excitation when the objects leave the ipsilateral hemifield (Omoto et al. 2017; Sun et al. 2017; Shiozaki and Kazama 2017). Thus, the developmentally defined anatomically parallel channels reflect differences in a visual modality of preference, receptive field structure, and temporal response properties.

The segregated visual information conveyed by the MeTu and TuBu neurons is transmitted onto the DALv2-derived ER-neurons (Fig. 2e; see all DALv2 CX neurons in Supp. File 2) (Omoto et al. 2017, 2018; Lovick et al. 2017; Hardcastle et al. 2021; Hulse et al. 2021). ER-neurons are the most abundant large-field neurons of the CX, with somata located in the anterior cortex dorso-laterally of the antennal lobes (Fig. 2a). They project postero-medially and branch at the level of the BU or lateral accessory lobe (LAL), into which each neuron extends a single dendritic proximal tuft. The distal branch extends medially along the lateral ellipsoid fascicle (LE, also called isthmus tract in Ito et al. (2014)) and arborizes circularly in the EB. The circular arbors of the ER-neurons give the EB its distinctive donut-like shape, within which the distribution of N-cad reveals 5 annular domains (Omoto et al. 2018). Individual ER-neurons connect the glomeruli in the different partitions of the BU to discrete annuli (Omoto et al. 2018). The innervation region in the BU (or LAL), N-cad domain, and the trajectory of the distal ER-neuron branches are defining characteristics for their nomenclature/classification. Based on these criteria, 11 morphological subclasses of ER-neurons have been described—all of which are post hoc discernable in the hemibrain despite the absence of N-cad reference (Omoto et al. 2018; Scheffer et al. 2020; Hulse et al. 2021). These 11 subclasses can be further broken down into 22 types using the synaptic information available in the hemibrain (Scheffer et al. 2020; Hulse et al. 2021).

The hemibrain connectomic analysis confirms that the selectivity for upstream TuBu partners by ER-neuron subclasses is quite stark. Much like the TuBu neurons, the dendritic tufts of ER-neurons are organized into compact glomeruli (except ER3a_a, ER3a_d; 8 neurons PBH). It is within these microglomerular complexes of overlapping TuBu and ER-neuron tufts that their synaptic connections are formed. Each ER-neuron subclass receives TuBu inputs exclusively from either DALcl1d or DALcl2d (Fig. 2e). ER-neurons with their proximal dendritic tufts located primarily in the LAL (21 PBH), as expected, do not receive TuBu inputs and are thus not a part of the AVP. These neurons and their inputs are discussed further below.

Despite converging onto neurons of the same lineage (DALv2 ER-neurons), parallel TuBu coding channels continue to remain anatomically segregated until their arrival in the EB (Fig. 2d). ER-neurons receiving input from the DALcl1d TuBu neurons innervate regions corresponding to the anterior (EBa) and outer central (EBoc) domains of the EB in the hemibrain (magenta in Fig. 2d). Complementarily, ER-neurons downstream of DALcl2d TuBu neurons innervate the more medially located inner central (EBic) domain (blue in Fig. 2d). Additionally, sparser and weaker innervation is visible in the inner posterior (EBip) domain/annulus (Fig. 2d). While it remains to be seen whether there are global neuronal markers that further subdivide this annulus, even within the EBip, the ER-neurons with different TuBu inputs maintain spatial segregation. EBip projecting ER-neurons downstream of DALcl1d and DALcl2d TuBu neurons occupy the more antero-medial and postero-lateral regions respectively (Fig. 2d). Finally, the DALcl2d TuBu to ER6 connections are reflected by the extremely sparse and weak labeling of the outer posterior (EBop) annulus in Fig. 2d. Thus, DALv2-derived neurons divide the EB into discrete annuli that reflect differences in input structure and modality.

Cell-type specific functional imaging experiments at the DALv2 leg of the AVP have been restricted to a few subclasses of ER-neurons, namely ER2 and ER4d in the BUs (Seelig and Jayaraman 2013) and ER4m in the BUa (ER4m circular processes in the EB have also been recorded) (Hardcastle et al. 2021). Consistent with their TuBu inputs, these neurons are tuned to small bright objects and the angle of polarization, respectively. While a systematic survey of the remaining ER-neuron subclasses is currently underway, the hemibrain does provide valuable insights into their putative functional interactions and the degree of influence on their postsynaptic partners (Hulse et al. 2021). Apart from the strong homo- and heterotypic interactions observed among the ER-neurons (Omoto et al. 2018; Hulse et al. 2021), their major targets in the EB are the so-called “compass neurons”. Compass neurons, or E-PG neurons, are a class of small-field neurons of the CX that integrate visual and proprioceptive cues to represent the animal’s instantaneous heading (Seelig and Jayaraman 2015; Fisher et al. 2019; Kim et al. 2019; Okubo et al. 2020; Haberkern et al. 2022). ER-neuron subclasses receiving inputs from the DALcl1d TuBu neurons consistently form stronger synaptic connections onto the compass network (Supp. Figure 2) (Omoto et al. 2018; Hulse et al. 2021). These synaptic contacts also tend to occur closer to putative spike initiation sites on the compass neurons—suggesting the dominance or preference for DALv2 inputs that are downstream of DALcl1d in shaping heading representation (Hulse et al. 2021).

Two subclasses, ER3p_a (downstream of DALcl2d-derived TuBu05) and ER5 (downstream of DALcl1d-derived TuBu06) stand out as clear exceptions to the above-described ER→E-PG connection pattern (Supp. Figure 2). Neurons of the latter subclass are the sole occupants of the EBa annulus and have been shown to play a role in the homeostatic regulation of sleep (Liu et al. 2016; Donlea et al. 2018; Omoto et al. 2018). Recent evidence also implicates two other ER-neuron subclasses downstream of the DALcl2d TuBu neurons, ER3d and ER3m, in the regulation of sleep and wake balance—suggesting functional interplay between the information inherited from the two developmentally distinct TuBu channels (via DALv2) (Aleman et al. 2021). The limited direct connectivity among these three subclasses suggests that this interaction is likely shaped by the convergence of signals within the compass network (Aleman et al. 2021; Hulse et al. 2021). Similarly, ER3p_a might be another example of such a convergence sub-network, shaping the balance between the fast and discrete nature of inputs inherited by the compass neurons from the DALcl1d channel as well as the slower more diffused responses of the DALcl2d TuBu_i_ neurons (via DALv2) (Omoto et al. 2017; Sun et al. 2017; Shiozaki and Kazama 2017).

In summary, the lineage-based organization of the AVP serves as a quintessential example of a circuit where individual developmental units tile the morphological and physiological feature space of inputs from the visual system. Each developmentally defined channel differentially influences the animal’s representation of its environment. The developmental trajectories followed by the constituent circuit elements sequentially divide each neuropil into distinct compartments (data not shown), positing interesting principles for genetically encoding stereotyped circuit assembly and potential mechanisms for deviation from the basic architecture.

#### Convergence of mechanosensory inputs via the LAL onto DALv2 ER-neurons: alternative input modalities

**Fig. 3:**
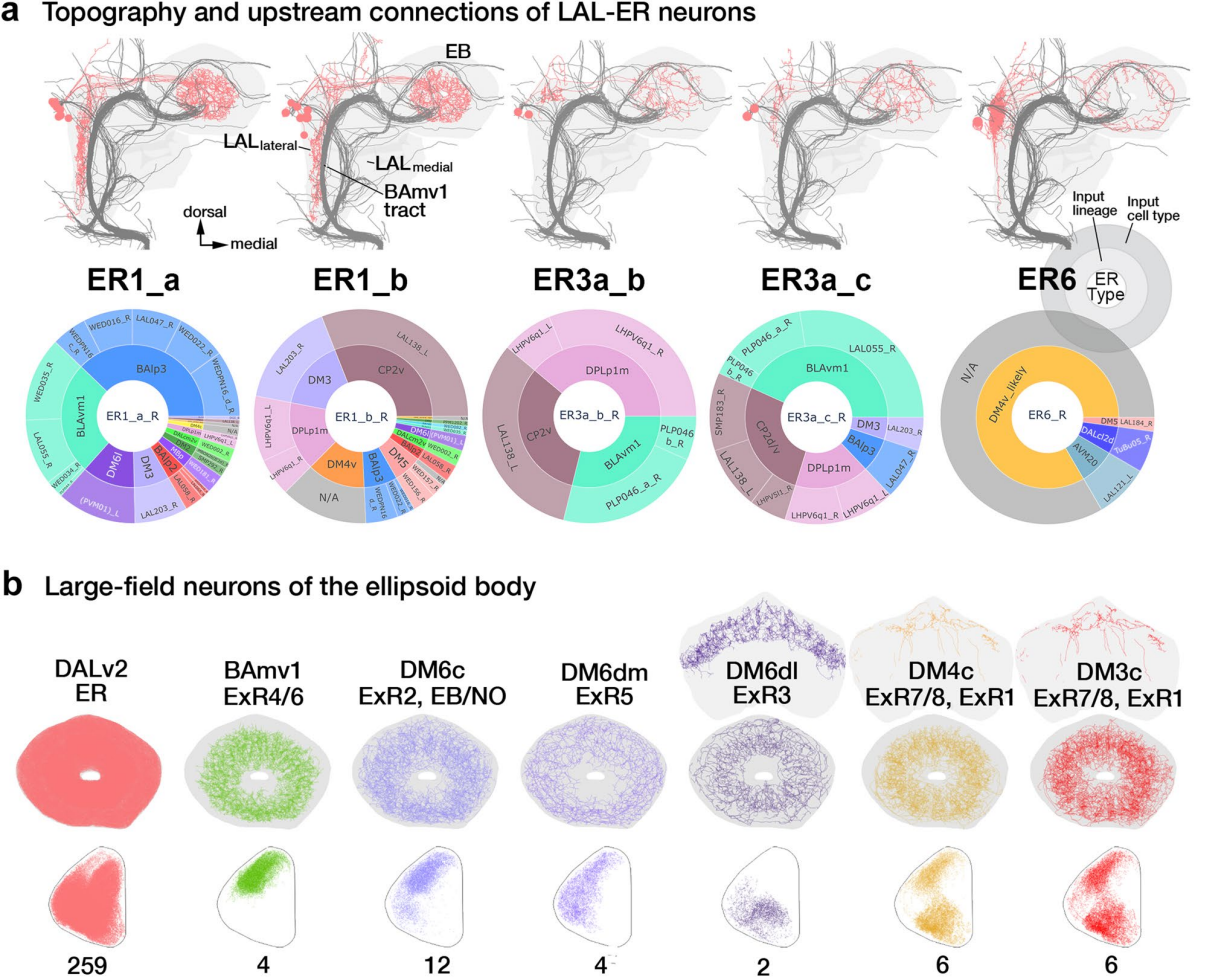
Organization and inputs to the ellipsoid body large-field networks apart from the anterior visual pathway. **a** Topography and upstream connections of DALv2 ER-neurons that receive input in the LAL, rather than the BU. Upper panels show plots of these five groups, including (from left to right) ER1_a, ER1_b, ER3a_b, ER3a_c, and ER6. Lower panels present sunburst plots showing external input to these neuron subclasses. Lineages containing neurons providing input to ER3a_b/c and ER1_a/b are indicated in the inner circle and the outer circle spells out individual neuron types included in the corresponding lineages. Sector size represents the fraction of total inputs provided to the ER-neurons by these lineages and the individual neuron types. **b** Plots of axonal arbors of ER-neurons (DALv2, left) and ExR neurons (defined by having connections outside the EB and BU), in the EB volume. Upper panels show frontal views of the EB, bottom row has a circular projection of the EB cross sections along the antero-radial axis (outline) with synapses of the constituent neurons (color coded) similarly collapsed onto a single plane. Refer to Fig. 2a for EB domain schematic. Names of lineages and specific types of ExR neurons contained within these lineages are given at the top and ExR neuron numbers (for both brain hemispheres) are shown at the bottom. Note that lineage DM6c has eight neurons that, while not included in the ExR category in the hemibrain, branch within the EB as well outside (FB, NO). Note also that ExR types ExR1, ExR3, ExR7, and ExR8 provide large-field input to specific FB layers, as indicated in the FB plots added at the top of these cell types

The bulb (BU) stands out as a structure dominantly innervated by the glomerular dendritic tufts of ER-neurons and acts as a conduit for visual information to the EB. The adjacently located neuropil compartment, the LAL, is another region that is innervated by dense dendritic arbors of a minority of ER-neurons (Omoto et al. 2018). These include neurons of the subclasses ER1, a subset of ER3a, and ER6 (Omoto et al. 2018; Hulse et al. 2021). A prominent distinguishing feature of these ER-neurons, apart from the LAL innervation, is the elongated and finely branched nature of their proximal tufts (Omoto et al. 2018). These tufts remain restricted to the lateral edge of the LAL (LAL_lateral_), a domain that is made apparent by the tract of the BAmv1 lineage passing through the neuropil (Fig. 3a).

Best characterized as a sensory convergence and premotor center, the innervation of the LAL by these ER-neuron dendrites suggests a potential role of these neurons in shaping the heading representation using distinct sensory cues that complement the AVP. Indeed, recent studies have described a role for ER1 and a subset of ER3a neurons in integrating wind-driven antennal displacement signals in the EB (Okubo et al. 2020). Although a functional and genetic characterization of the entire pathway to the periphery remains incomplete, Hulse et al. (2021) attempt to trace putative input circuitry formed by these neurons.

Narrowing our analysis to the first-order upstream partners—we immediately find, in sharp contrast to the AVP, a convergence of signals from multiple lineages onto these ER-neurons (Fig. 3a). ER3a_b and ER3a_c neurons receive the most prominent inputs from the lineages BLAvm/SLPa&l1, CP2v, and DPLp1/LHp2 (Fig. 3a). These inputs are relatively weak, ranging between 20 and 50 synapses per input neuron type (to the entire ER3a population). ER3a_c receives additional weak inputs from individual neuron types of BAlp3 and DM3 (Fig. 3a). The two types of ER1 neurons, ER1_a and ER1_b, have a more developmentally diverse input profile than ER3a (Fig. 3a). They also differ more starkly among each other compared to the aforementioned ER3a types. ER1_a receives the strongest inputs from BAlp3 and BLAvm. The strongest ER1_b inputs belong to CP2v, DM3, and DPLp1 (Fig. 3a).

The CP2v-derived LAL138, putatively corresponding to the wedge-LAL-LAL (WL-L) neuron, as well as the DPLp1-derived LHPV6q1 (likely the wind-direction sensitive wedge projection neuron—WPN) have been shown to be responsive antennal displacements (Suver et al. 2019; Okubo et al. 2020). These prominent connections ascertain the role of ER1_b, ER3a_b, and ER3a_c in forming the wind mechanosensory input channel into the EB (Hulse et al. 2021). ER1_a is devoid of CP2v inputs and is only weakly (19 synapses) connected to DPLp1. This highlights the functional differences between the two morphologically similar ER1 neuron types that has not been observed at the light-microscopy resolution (Omoto et al. 2018). The ER1_a pathway might represent a complementary mechanosensory stream into the EB through the BAlp3 lineage.

Unlike what has been shown for other insects, the antennal mechanosensory dependence of behaviors in flies is not as extensive (Fuller et al. 2014; Mamiya and Dickinson 2015). The small size of the ER1 and ER3a populations and the convergence of inputs generated by a relatively large number of different lineages onto them might reflect an evolutionary appropriation of remnant streams of direct and indirect mechanical signals from the Johnston’s Organs via the antennal mechanosensory and motor center (AMMC), wedge (WED), and antler (ATL)—compartments targeted by BLAvm, CP2, and DPLp1.

#### The EBop: lateral inputs into the recurrent circuitry formed by ER6 and the CX small-field neurons

With large dense arbors in the dorso-lateral LAL, a domain called the gall (GA), the ER6 neurons are the only ER-neuron population that targets the EBop annulus. This domain is one of the principal recipients of columnar neurons reaching the EB. Given the small number of ER6 neurons (2PBH), their innervation in the EBop is relatively weak (Fig. 3a, b). The limited functional evidence as to their role in this domain comes from the optogenetic activation of drivers targeting this population (Franconville et al. 2018). These recordings confirm their inhibitory nature—as are all other DALv2 ER-neurons.

Most inputs to ER6 neurons outside the EB are from the CX columnar neurons targeting the GA—thus forming a recurrent circuitry between the small- and large-field networks of the EB (Hulse et al. 2021). ER6 neurons do receive non-CX inputs in the GA as well, which almost exclusively originate in the contralateral (left) hemisphere. Despite missing the tract entry portal (the spatial coordinate where the tract bundle enters the neuropil volume) and the proximal part of the arbors, based on the commissural trajectory taken by these neurons to reach the GA (right), we think that they very likely belong to the DM4v hemilineage (388 synapses; Fig. 3a). Despite not being able to identify any of these neurons, we see that the ER6 population, like the ER-neurons of the AVP, also displays a strong lineage bias in lateral upstream inputs—further investigation into which will require datasets that include both hemispheres.

#### Extrinsic ring neurons: diverse developmental origins of modulatory inputs to the central complex

The ExR neurons are more developmentally diverse than the ER-neuron population. Genetic studies have identified four ExR neuron types, to which the hemibrain has added four more (Omoto et al. 2018; Hulse et al. 2021). Named numerically ExR1-8, these neurons originate from the BAmv1 (Supp. File 3), DM6 (Supp. File 4–6), DM4 (Supp. File 7, 8), and DM3 (Supp. File 9) lineages (Fig. 3b).

The ExR1 neurons (2PBH), more commonly referred to as the Helicon cells, are derived from DM3 (1PBH) and DM4 (1PBH) lineages (Donlea et al. 2018; Omoto et al. 2018). Not only are these lineages the source of the columnar network of the CX, but this developmental motif of the same neuron type originating from multiple neuroblasts is also characteristic of the columnar neurons. Despite being a “large-field” neuron, the ExR1 neurons anatomically (by interconnecting the FB and EB) and developmentally resemble the small-field population. These two lineages, also give rise to ExR7 and ExR8 neurons in a similar fashion (Fig. 3b). More typical developmental profiles are seen among the ExR4 and ExR6 (BAmv1) as well as the ExR2 (DM6c; Supp. File 5), ExR3 (DM6dl; Supp. File 6), and ExR5 (DM6dm; Supp. File 4) neurons (Fig. 3b).

Like the DALv2 neurons, ExR neurons also innervate the EB in a spatially restricted manner. ExR1 neurons occupy the EBa and EBic annuli. ExR2, ExR4, ExR5, ExR6, ExR7, and ExR8 neurons densely innervate the EBop. ExR2 and ExR5 also extend sparse arbors into the EBoc. ExR3 neurons primarily occupy the EBic (Fig. 3b). The EBop stands out as a domain that is very weakly occupied by the ER-neurons and displays a strong preference for the ExR population.

ExR neurons have large arbors which reflect in their extremely broad connectivity patterns. The inputs and output motifs of ExRs are described at length in Hulse et al. (2021). Overall, their large widespread morphology is correlated with a likely modulatory role in the CX circuitry. Functional studies of this population are limited—but most of them affirm broad activity-modulating roles for these neurons. ExR1 (Helicon) neurons have been shown to be involved in regulating sleep homeostasis (Donlea et al. 2018). The ExR2 neurons, PPM3 dopaminergic neuron population, promote arousal associated with circadian behavior peaks as well as ethanol exposure (Kong et al. 2010; Omoto et al. 2018; Liang et al. 2019). They have also been speculated to play a role in training the activity profiles of the columnar neurons with respect to the visual field as well as modulating the amplitude of visual inputs to the CX (Hulse et al. 2021; Grover et al. 2022; Frighetto et al. 2022; Fisher et al. 2022). ExR3 neurons are serotonergic (Omoto et al. 2018) and have also been implicated in the regulation of sleep architecture (Liu et al. 2019). Beyond this, the ExR neurons are a mystery.

### Every lineage has a story: modular organization of the inputs and intrinsic circuitry of the protocerebral bridge

The posterior-most neuropil compartment of the central complex (CX) is the handlebar-shaped protocerebral bridge (PB). As its name suggests, the PB acts as an anatomical and functional bridge between the small-field networks of the ellipsoid body and the fan-shaped body (Wolff et al. 2015; Wolff and Rubin 2018; Turner-Evans et al. 2020). It is organized into nine discrete compartments per hemisphere, called glomeruli, wherein signals get reformatted and propagated forward (Turner-Evans et al. 2020). These signal transformations are achieved through a combination of small- and large-field elements interconnecting individual glomeruli in unique configurations (Wolff et al. 2015; Turner-Evans et al. 2020).

**Fig. 4:**
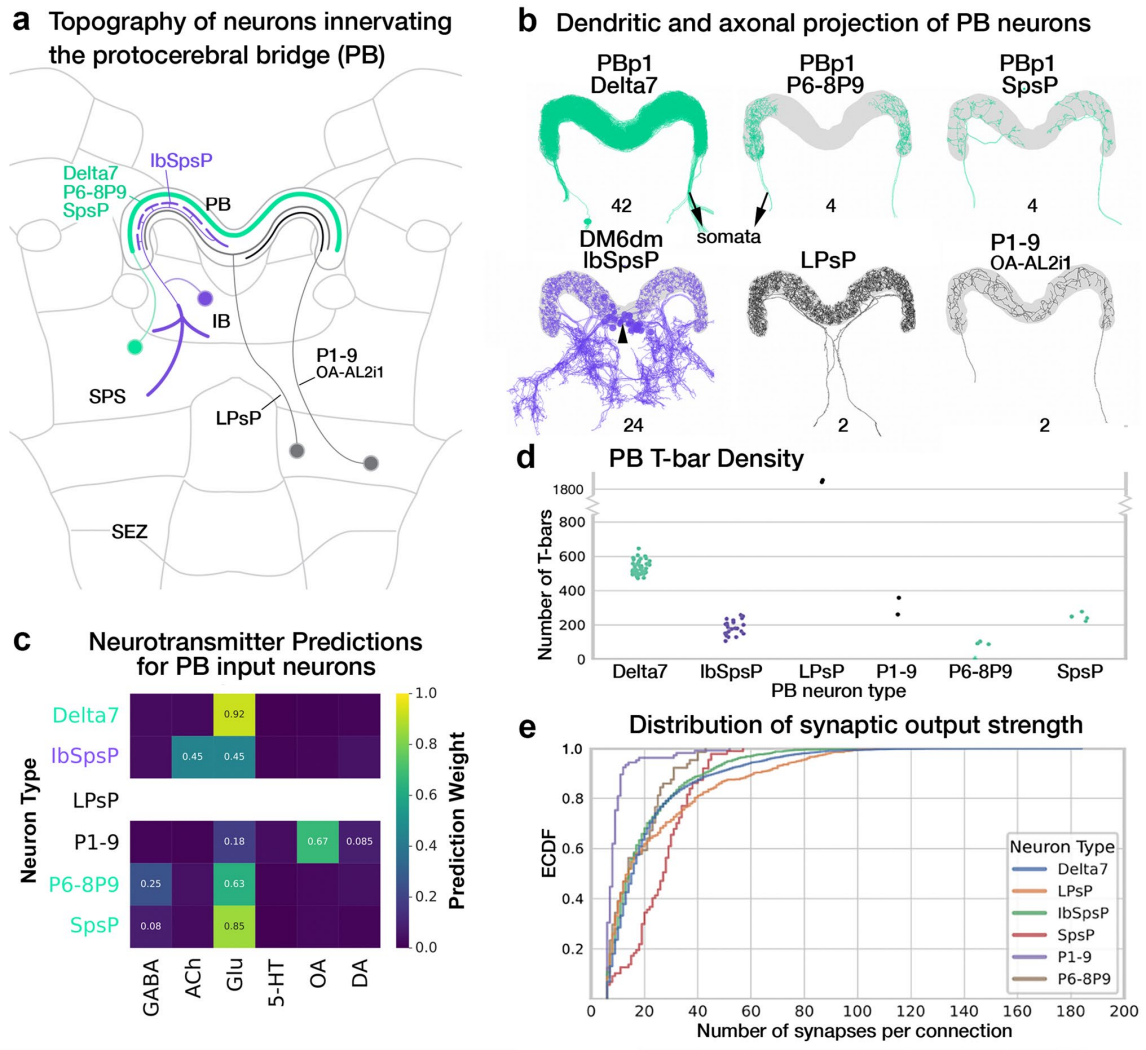
The modular developmental architecture of the protocerebral bridge. **a** Schematic frontal section of the brain at a posterior level, visualizing the PB and surrounding neuropil compartments, as well as lineages innervating the PB. PBp1 provides large-field neurons branching throughout the PB but has only very sparse connections outside this compartment (mostly intrinsic neuron; see also Fig. 1a). IbSpsP neurons form a group that most likely belongs to the large lineage DM6 (tract DM6dm). These neurons tile the PB with spatially restricted axonal arbors and have extensive dendritic arbors in the inferior bridge (IB) and superior posterior slope (SPS). Two individual neurons with large-field input to the PB, LPsP and P1-9/OA-AL2i1, belong to as yet not identified lineages with cell bodies in the subesophageal zone (SEZ). **b** Plots of PB-innervating neurons schematically shown in (**a**), presented in anterior view. Names of lineages and specific types of PB neurons contained within these lineages are given at the top of each image and the corresponding neuron numbers (for both brain hemispheres) are shown at the bottom. Note that somata are shown only for IbSpsP neurons (arrowhead). For all other neuron types, somata are outside the hemibrain volume. **c** Heatmap showing neurotransmitter prediction for different neurotransmitters listed along the horizontal axis. **d** Number of T-bars formed by each neuron in the PB color-coded by lineage and organized by individual neurons. Of note, P6-8P9 and SpsP neurons, in addition to being numerically smaller populations also form fewer T-bars per neuron in the PB compared to their PBp1 sister population Delta7. Dopaminergic LPsP neurons form over three-fold higher number of T-bars in the PB than Delta7. **e** ECDF of the pairwise connectivity strength in the PB formed by the different neuron types. P1-9/OA-AL2i1 octopaminergic neurons form the fewest and weakest connections. Of the PBp1 population, Delta7 neurons (blue) form the most and strongest connections

Lateral inputs to the PB are relatively few in number and morphology (Fig. 4a, b) (Lin et al. 2014; Wolff et al. 2015; Wolff and Rubin 2018). All but four neurons (2PBH) belong to two discretely identifiable tracts corresponding to the lineages DM6dm (Supp. File 4) and PBp1 (Supp. File. 10) (Fig. 4b). The four outliers are fragmented, with the proximal parts of their tracts falling outside the hemibrain volume (Fig. 4a, b). However, aided by morphology in the PB and neurotransmitter predictions, we were able to ascertain their individual identities and putative developmental origins (Fig. 4a–c) (Eckstein et al. 2020; Scheffer et al. 2020).

The only dominant source of inputs to the PB from lateral neuropils is the rather atypical-looking IbSpsP (PB_G2-9_.b-IB.s.SPS.s from Wolff et al. (2015)) population. These neurons have never been observed in any lineage clone (Ito et al. 2013; Yu et al. 2013; Wong et al. 2013). However, they fall within the bundle which we independently annotated as being a part of the DM6 lineage—displaying the utility of the hemibrain in filling gaps in the genetic characterization of brain development (Supp. Figure 1). IbSpsP somata are located in the posterior cortex, ventral to the PB, on either side of the midline (Fig. 4b). Their projections traverse along the DM6dm tract, extending ventro-laterally and branching at the postero-lateral surface of the inferior bridge (IB). The distal dendritic tufts form a unique slender trident structure—extending spiny profiles medio-ventrally and dorso-laterally along the posterior surface of the IB, as well as ventro-laterally along the posterior surface of the superior posterior slope (SPS; Fig. 4a, b). The axons extend dorsally towards the PB, with each neuron innervating only one or two glomeruli. The spread and synaptic profiles of the dendrites of IbSpsP neurons outside the CX are typical of large-field neurons, while the PB innervation pattern resembles that of the small-field neurons. These derivatives of DM6, thus, represent a structural hybrid between the two major classes of CX neurons.

The neurons of PBp1 are not without their own oddities. Their somata are missing from the hemibrain imaged volume (Fig. 4b). Genetic clones and drivers targeting these populations reveal two distinct clusters of somata in the posterior cortex at the level of the PS (Yu et al. 2013; Lu et al. 2022). Tracts originating from each cluster project medio-dorsally and coalesce before continuing dorsally towards the lateral edges of the PB (Fig. 4b). Of the three neuron types formed by this lineage, both clusters contain the Delta7 neurons, while SpsP and P6-8P9 neurons exclusively belong to the dorsal and ventral clusters, respectively. Within the PB, neuron types SpsP (PB_G1/2–9_.b-SPSi.s; 2PBH) and P6-8P9 (PB_G6-8_.s_G9_.b; 2PBH), remain restricted to the glomeruli of the ipsilateral hemisphere instead of spanning the entirety of the PB (Fig. 4b) (Wolff et al. 2015). The latter neuron type displays further segregation of morphological and synaptic profiles across the glomeruli. Predominant output terminals, or boutons, are restricted to the 9th glomerulus, with spiny dendritic profiles occupying the 6th, 7th, and 8th glomeruli (Wolff et al. 2015). The most numerous neuron type within PBp1, Delta7 (21PBH; PB18.s-GxΔ7Gy.b in Wolff et al. (2015)), much like a typical large-field neuron spans the entire PB (Fig. 4b). However, amid the dendritic branches that span the entire PB, each neuron displays two (on occasion three) clusters of output bouton terminals spaced seven glomeruli apart (Wolff et al. 2015). As a population, these varicose profiles of individual Delta7 neurons innervate all PB glomeruli. Interestingly, other than the sparse arbors of the SpsP neurons (2PBH) in the SPS, the entire projection envelope of PBp1 is contained in the PB—a strong neuropil selectivity not displayed by any other lineage (Yu et al. 2013).

The two neurons (1PBH) annotated as LPsP by Scheffer et al. (2020), correspond to the PB.b-LAL.s-PS.s. neurons described by Wolff et al. (2015) (CIVP in Lin et al. (2014), T1 in Mao and Davis (2009)) (Fig. 4b). These neurons have also been previously observed in genetic clones derived using drivers that target dopaminergic neurons—an indicator of their neuromodulatory role (FlyCircuit clone: TH-F-000048; Chiang et al. (2011)). Their cell bodies are located in the anterior cortex, ventro-medial of the antennal lobes (Chiang et al. 2011; Lin et al. 2014; Wolff et al. 2015). The tract extends posteriorly and branches laterally, extending proximal tufts into the ipsilateral LAL and Posterior Slope (PS). The distal branch extends towards the posterior surface of the brain, turns sharply dorsally, and bifurcates before innervating the PB (Fig. 4b). The stochastic labeling approach used by Chiang et al. (2011) to generate the clone, the absence of such a neuron in any neuroblast lineage clone catalogued to date, and the deviation from any major tract in the vicinity (BAm cluster) suggests that these neurons are embryonically born (Yu et al. 2013; Lovick et al. 2013; Wong et al. 2013; Hartenstein et al. 2017; Kendroud et al. 2018). They are likely functional in the larval brain and transdifferentiate during pupation to be incorporated into the PB in the adult brain.

The synaptic profiles of the other two neurons, annotated as P1-9, are strongly predicted to be octopaminergic (Fig. 4c). Along with their dispersed and varicose morphology in the PB, the neurotransmitter predictions suggest that these fragments belong to the OA-AL2i1 neurons described in Busch et al. (2009). Entering the PB laterally, the fragments reconstructed in the hemibrain are a small part of the extensive and diverse branching displayed by this neuron type (Busch et al. 2009; Wolff and Rubin 2018). Their somata are also located in the anterior cortex, ventro-medially of the antennal lobes—with the branches extending into the PS, ventromedial protocerebrum, inferior protocerebrum, lobula, and medulla (Busch et al. 2009). Like the LPsP neurons, the OA-AL2i1 neurons also do not appear in any neuroblast lineage clone, suggesting possible embryonic origins (Yu et al. 2013; Lovick et al. 2013; Wong et al. 2013; Hartenstein et al. 2017; Kendroud et al. 2018).

In addition to the structural differences in the PB, neurons from each of the aforementioned classes also display functional differences. The restriction of the PBp1 projection envelope to the PB posits the role of this lineage in forming the interneuron network to regulate local activity. Indeed, functional imaging experiments have shown that Delta7 neurons are involved in stabilizing and reformatting signals in the small-field network (Turner-Evans et al. 2020). They serve as inhibitory neurons that restrict small-field neuron activity to specific glomeruli—critical to the appropriate representation of spatial information within this network (Fig. 4c) (Turner-Evans et al. 2020). The P6-8P9 neurons are likely inhibitory (predicted as GABAergic or Glutamatergic) and might serve a similar role (Fig. 4c). Finally, the SpsP neurons stand out as an exception within this group. Although they do receive external inputs in the SPS, their synaptic contribution—which we measured as the number of T-bars in the PB (Fig. 4d) and distribution of pairwise connection strengths (Fig. 4e)—is smaller than that of the Delta7 neurons. They likely respond to regressive optic flow and have been shown to influence the amplitude of signals primarily in the PFNd small-field population (Lu et al. 2022; Lyu et al. 2022). This small sphere of influence leads us to attribute PBp1 as primarily building the intrinsic network of the PB in *Drosophila* (see section on evolution and discussion). Complementarily, DM6-derived IbSpsP neurons function as conduits of lateral velocity signals to a broader diversity of PB small-field and PBp1 neurons (Scheffer et al. 2020; Lu et al. 2022). Lacking functional insight into the two neuromodulatory populations, LPsP and P1-9 (OA-AL2i1), we can speculate their role in modulating or learning spatial representation patterns based on the behavioral state of the animal.

Owing to the restricted imaging volume of the hemibrain, the major upstream partners of the PB neurons (atypical large-field neurons) are significantly fragmented. This precluded our ability to trace input channels and determine developmental connectivity logic. However, even at the level of the PB, our analysis has revealed stereotypic developmental organizational principles (intrinsic reformatting via PBp1, lateral inputs via DM6, and two likely embryonically born sources of neuromodulatory inputs). The PB is thus a clear example of developmental segregation of discrete circuit elements—a neuropil with modular origins (see also discussion; Fig. 4a).

### Of lineages and layers: the diverse structural and functional constituents of the fan-shaped body

**Fig. 5:**
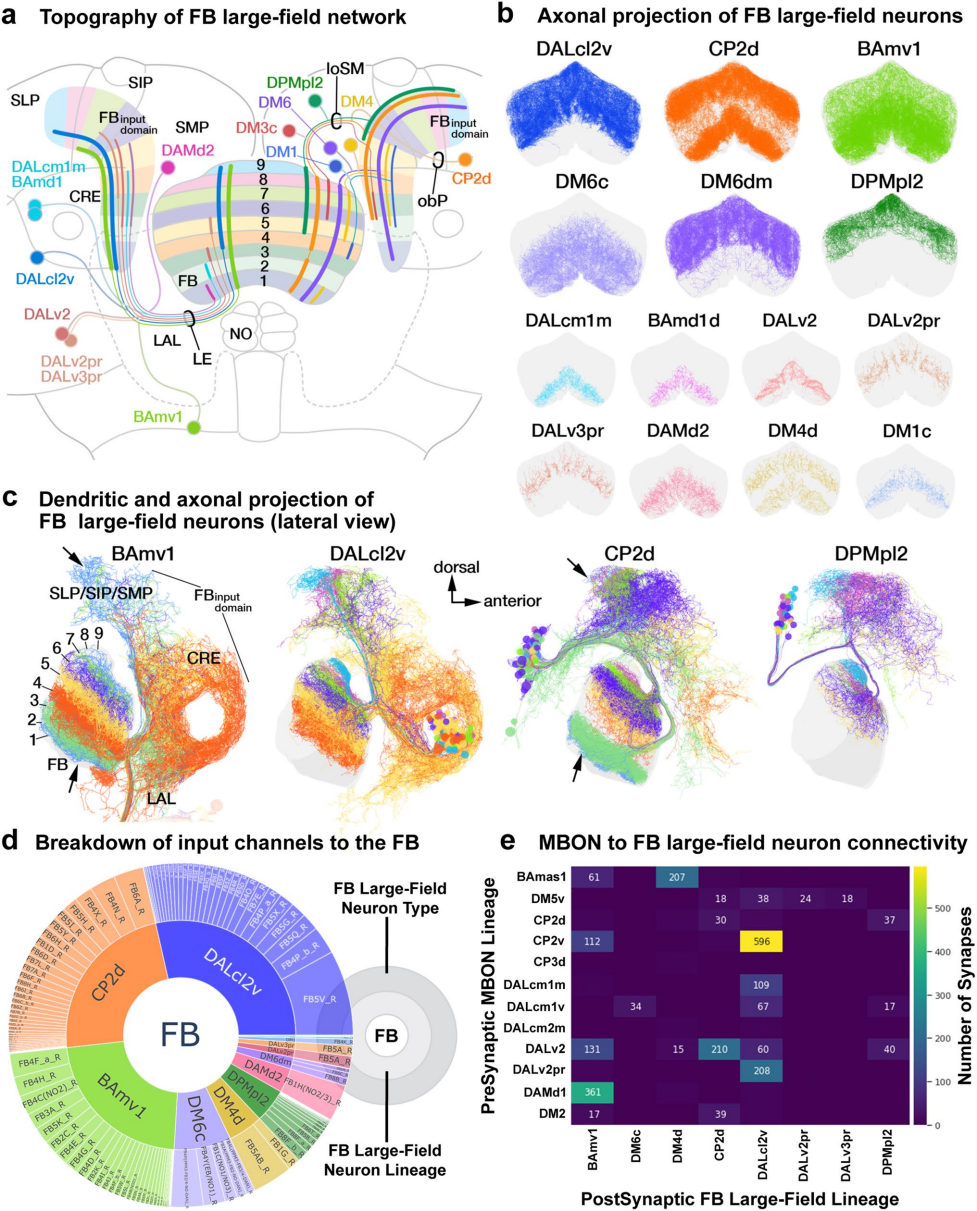
Diversity and anatomical tiling properties of the large-field neuronal constituents of the fan-shaped body (FB). **a** Schematic frontal section of a brain hemisphere at central level, visualizing the FB and surrounding neuropil compartments, as well as lineages with neurons that provide large-field arbors in the FB. Lineages with somata located in the anterior brain are shown on the left and those with somata in the posterior brain on the right. Somata clusters are represented by colored circles with the names of the corresponding lineage next to them. Position of circles roughly coincide with the location of somata clusters in the brain. The projection envelope of a lineage, rendered in the same vivid color as its soma cluster, is divided into a dendritic part that innervates the fan-shaped body input domain (FB_ID_) located in the lateral protocerebrum (SLP/SIP/SMP/CRE/LAL), and an axonal part covering certain layers of the FB. For example, neurons of DPMpl2 (dark green) project to the FB dorsal layers 5–9 and have dendrites in the posterior half of the FB input domain. Distinguished are five “major” lineages that contribute the large majority of FB large-field neurons (BAmv1, DALcl2v, CP2d, DPMpl2, DM6), represented by thick lines for input/output, from the remainder of “minor” lineages shown by thin lines. A second system of muted colors, independent of the vivid colors marking lineages and their projections, is employed to visualize the topographical correlation between input domain and FB output layer. Neurons innervating dorsal layers of the FB tend to have dendrites at more posterior locations in the FB_ID_ (see also panel **c**). **b** Plots of axonal arbors of FB-innervating neurons schematically shown in (**a**), presented in anterior view. ExR neuronal arbors are rendered in Fig. 3. **c** Plots of complete arbors of four of the five major lineages (BAmv1, DALcl2v, CP2d, DPMpl2) in lateral view (anterior to the right, dorsal up). Within each lineage, neurons ending in a specific FB layer are rendered in the same color. For all four lineages, neurons innervating the more dorsal layers (purple-light green-magenta-cyan) have dendritic arbors in the posterior FB_ID_ (SLP, SIP). Axonal arborization in the central FB layers (4, 5) correlates with dendritic arborization in the CRE; axonal arborization in the ventral layers (2, 3) with dendritic arborization in the antero-ventral FB input domain (SMP, posterior CRE, LAL). An exception is layer 1, innervated by a group of BAmv1 and CP2d neurons, that is correlated with dendritic endings in the most posterior FB_ID_ (arrows). **d** Sunburst plot visualizing lineages of origin of large-field FB neurons (inner circle) and their break-down into individual neuron types as defined in the hemibrain (outer circle). The size of each sector depicts the number of input synapses onto these neurons/lineages from lateral neuropil compartments. **e** Heatmap depicting input from mushroom body output neurons (MBONs) onto FB large-field neurons. Overall, large numbers of neurons of extremely diverse lineage associations (more than 30 lineages of origin; not shown) provide input to the large-field dendrites in the FB_ID_. However, if filtered for specific subtypes of input neurons, like the MBONs presented in this heatmap, only a few lineages (e.g., BAmas1, CP2v, DALv2, DAMd1) provide the bulk of synapses to select FB large-field neuron groups. For abbreviations see Table 1

The most developmentally diverse and numerically complex neuropil compartment of the central complex (CX) is the fan-shaped body (FB; Fig. 1f, g; Fig. 5a, b). It is organized into nine layers along the dorso-ventral axis (Wolff et al. 2015), each of which constitutes the innervation domain of a unique set of large-field neurons (Fig. 5a, b). The orthogonal axis is also anatomically specified into nine columns, as evidenced by the nine protruding “teeth” along the ventral surface of the FB (Wolff et al. 2015). The synaptic profiles of individual small-field neurons that remain restricted to the ventral layers and collectively tile the medio-lateral axis, corroborates this segregation (Wolff et al. 2015; Hulse et al. 2021). More dorsally, however, no clear anatomical markers delineate columnar boundaries. Concomitantly, clonal labeling of small-field neurons innervating these layers reveals considerable overlap along the edges of the “adjacently” projecting neurons (Wolff et al. 2015). Furthermore, the hemibrain dataset shows that a few small-field neuron types deviate in the number of columns they carve out in the FB (Hulse et al. 2021). The neuronal constituents of the FB, in effect, form a complex grid-like network with variable dimensions along the dorso-ventral axis.

The large-field neurons constituting the input network to the FB, interconnect a large domain within the dorsal and anterior protocerebrum, which we call the “fan-shaped body input domain” (FB_ID_), to the different FB layers in a topographically ordered fashion (Fig. 5a, b). The FB large-field neurons belong to one of twelve lineages—five “major” lineages (DALcl2v, CP2d, BAmv1, DM6, and DPMpl2) forming most of the neurons and the remaining “minor” lineages (BAmd1d, DALcm1m, DALv2, DALv3, DAMd2, DM1, DM3, and DM4) contributing only one or two neurons per hemisphere. Neurons of each lineage preferentially innervate a subset of the nine layers, with the 5th and 9th layers standing out as the most and least diverse, respectively (Fig. 5b; Supp. Figure 3). Additionally, one pair of large octopaminergic neurons (OA-VPM3), originating in the SEZ also broadly innervates the FB.

#### Developmental diversity and structural features of the FB large-field ensemble

The ventral hemilineage of DALcl2 (DALcl2v; blue; Supp. File 11), constitutes the largest number of FB large-field neurons (Right = 70; Left = 72; Fig. 5a–c). With somata located in the anterior cortex, medially of the lower unit of the AOTU, DALcl2v neurons extend their fibers postero-ventrally under the peduncle (PED) before turning medially and joining the lateral ellipsoid fascicle (LE; Supp. Figure 1). They branch at the level of the superior bulb (BUs), from where the proximal branches extend dorsally and arborize densely in the CRE, SMP, SLP, and SIP. A few neurons extend proximal branches ventrally to form tufts in the dorsal part of the LAL (Fig. 5c). The distal branches, while following the LE, segregate into a distinct dorsal and ventral subpopulation (Fig. 5c, Supp. Figure 1). Both subpopulations proceed along the anterior surface of the FB, with the former being positioned at the level of the dorsal tip of the EB. From there, each neuron extends multiple branches posteriorly into the FB neuropil. Interestingly, while the dorsal subpopulation of DALcl2v collectively spans the entire FB projection envelope of DALcl2v (right hemisphere = 45), neurons of the ventral subpopulations mainly innervate the 4th and 5th layers (right hemisphere = 19). A few neurons sparsely innervating the 6th (right = 1), 7th (right = 3), and 8th (right = 2) layers are also contained in the ventral subpopulation (Supp. Table 3).

The dorsal hemilineage of CP2 (CP2d; orange; Supp. File 12) is a close second, contributing 131 FB large-field neurons across both hemispheres (among other neurons; Fig. 5a–c). The somata of these neurons are located in the posterior cortex, ventro-laterally of the calyx (CA). Their fibers project antero-medially across the peduncle in front of the CA. Their conspicuous fiber bundle forms the oblique posterior fascicle (obP; Supp. Figure 1). CP2d neurons have a branch point slightly posterior to the vertical lobe of the mushroom body (MB), along the lateral edge of the SMP. Proximal dendritic tufts are densely packed in this corner of the SIP/SMP. A few neurons extend proximal branches ventrally and arborize in the LAL and CRE. The distal branches continue as a tight bundle that bends sharply medio-ventrally towards the FB. The bundle splits into two components at the dorso-lateral edge of the FB, with one half extending medially (dorsal subpopulation) and the other continuing ventrally towards the midline (ventral subpopulation) (Supp. Table 4). Neurons of the dorsal subpopulation project directly into the FB, exclusively innervating the 4–7th layers. The bundle formed by the ventral subpopulation continues anteriorly, makes a 180° turn at the anterior surface of the EB to then project backwards through the EB canal towards the FB. Most CP2d neurons of the ventral subpopulation innervate the 1st and 2nd layers. A few neurons bi- or tri-furcate and move dorsally along the anterior surface of the FB before primarily innervating the 5th (right = 2), 6th (right = 1), 7th (right = 2), and 8th (right = 6) layers (Supp. Table 4). The dorsal subpopulation thus maintains exclusivity for the 5th layer, while the ventral subpopulation retains exclusivity for the 1st, 2nd, and 8th layers. This arrangement of CP2d neurons leaves the 9th layer almost devoid of synaptic profiles. Innervation into the 3rd layer is also relatively sparse, resulting in a visible “gap” in the hemibrain and genetic clones of this hemilineage (Fig. 5b; Fig. 1d, d’; Supp. Figure 3a–c).

The BAmv1 neuroblast lineage is the broadest developmental unit of the CX large-field network (Fig. 1g; Supp. File 3). Their somata are located ventrolaterally of the antennal lobes (AL; Supp. Figure 1) and project fibers along a large fascicle, called the longitudinal ventromedial fascicle (loVM; Supp. Figure 1) (Lovick et al. 2013). This bundle then splits into three major components. The first continues posteriorly into the ventromedial cerebrum. The second turns laterally and extends towards the ventrolateral protocerebrum. The third, which contains all the CX-directed neurons of BAmv1, turns upward and then medially through the LAL towards the CX—forming 120 (across both hemispheres) FB-projecting neurons. The primary branch point of BAmv1 neurons finds itself at the postero-dorsal surface of the LAL. Dendritic branches project upward or forward, targeting the SLP, SIP, SMP, CRE, and LAL. Distal axonal branches continue medially, forming the posterior component of the LE, and proceed towards the antero-ventral surface of the FB. From here, terminal branches radiate dorsally, collectively spanning the entire dorso-ventral axis of the FB, with the highest synaptic density in the 4th layer (Fig. 5c; Supp. Figure 3a, c). BAmv1 also contains an interesting set of FB neurons that extend arbors to multiple layers with large gaps between them (FB1I and FB1J) (Scheffer et al. 2020). These bi-layered neurons interconnect the 1st layer with the 7th and 8th layers, suggesting a functional relationship between the ventral and dorsal extremes of the neuropil (Fig. 5c; Supp. Figure 3b).

DM6, another major CX contributor, represents one of eight type II lineages, which generate more neurons and extend more tracts than the “typical” type I lineages. DM6 forms six unique tracts, three of which contain neurons that innervate the FB—DM6dm (Supp. File 4), DM6c (Supp. File 5), and DM6dl (Supp. File 6) (Fig. 5b). DM6 somata are located in the posterior cortex, near the edge of the protocerebral bridge (PB). The DM6dm bundle extends anteriorly and dorsally along the posterior dorsal surface of the FB, from where fibers of the large-field neurons curve ventrally and arborize predominantly in the dorsal layers of the FB. A few neurons extend sparse synaptic profiles well into the 2nd layer (Fig. 5b; Supp. Figure 3a). Most DM6dm neurons (the only lineage except PBp1 to do so) are intrinsic to the FB (FB4Z, FB5R, FB5S, FB5U, FB6J, FB6L, FB7D, and FB7J), lacking external arbors, and predominantly innervate the 5th and 6th layers, with weaker innervation in the 4th, 7th, and 8th layers. A subset of DM6dm neurons gives off proximal tufts that extend into the wedge (WED).

The DM6c cluster (Supp. File 5) tract enters the neuropil ventral to the DM6dm entry portal. It extends anteriorly along the medial equatorial fascicle (MEF). FB projecting neurons in this bundle turn sharply medially at the ventro-lateral edge of the FB. From here, the dendritic arbor extends densely into the CRE and LAL (as far posterior as the WED) and sparsely into the SMP. Distal branches of DM6c innervate the ventral layers of the FB, with weak synaptic profiles extending only as far as the 6th layer (Fig. 5b; Supp. Figure 3a). All but one type (FB4L, 2PBH) of DM6c neurons also extend a third set of branches bilaterally into the noduli (NO). The only FB innervating constituents of the DM6dl (Supp. File 6) tract are the ExR3 serotonergic neurons (as discussed in the ellipsoid body section).

The last of the major FB large-field lineages is DPMpl2 (Supp. File 13). With somata in the posterior cortex, the DPMpl2 tract enters the neuropil as part of the superior medial longitudinal fascicle (loSM) (Lovick et al. 2013). Neurons extend proximal tufts that extend into the SLP, SIP, and SMP. The distal branches continue medio-ventrally towards the dorso-lateral edge of the FB. DPMpl2 innervation in the FB targets the dorsal layers (5–9th; Fig. 5b, Supp. Figure 3a).

Of the minor lineages, the most distinct are the neurons of DALv2 (Supp. File 2). Traversing and branching alongside the ER-neurons, the FB innervating component of DALv2 enters the EB canal and continues posteriorly. The neurons begin bifurcating as they reach the posterior part of the canal and proceed to innervate the FB—predominantly targeting the 3rd layer (Fig. 5b; Supp. Figure 1). A single neuron type in this lineage, FB3B, also shares innervation at the boundary of the EBip and EBop much like ER1 neurons. The proximal tufts of the DALv2 FB neurons extend one set of branches into the LAL, resembling ER3a_b, ER3a_c, and to a lesser extent ER1 (Supp. Figure 4). They also form a second set of slender branches towards the SMP. In addition to the dendritic morphology, these neurons also share putative inputs encoding antennal displacements with the ER3a and ER1 subclasses—suggesting an extension of the mechanosensory role of DALv2 into the FB (Supp. Figure 4).

Ventrally of DALv2 are two bundles formed by the embryonically born (primary) DALv2/3 neurons (Hartenstein and Kandimalla, in preparation; DALv2pr and DALv3pr; Supp. File 14, 15, respectively)—each bundle containing one neuron per hemisphere innervating the FB. In line with the notion of their embryonic origins, neurons with this morphology cannot be detected in MARCM clones (which tend to selectively visualize larval/pupal-born neurons i.e. secondary neurons). All four FB (2PBH) neurons in the DALv2pr (Supp. File 14) and DALv3pr (Supp. File 15) bundles were classified as FB5A by Scheffer et al. (2020). These neurons extend proximal dendritic branches into the LAL and distal axonal branches into the 5th layer of the FB (Fig. 5b). Neurons from the two bundles differ primarily in the extent of the neighboring layers that they span, with DALv2pr and DALv3pr FB5A predominantly extending large varicose profiles ventrally and dorsally, respectively. The disparate developmental origins and the unique morphology suggest that these neurons might in fact be more “different” than currently suspected. Like DALv2 FB neurons, they also appear to incorporate mechanical or movement-related cues (via PFL2 and PFL3—CX output channels). They are additionally targeted by LC33b neurons—reminiscent of the DALv2 ER-neurons which also convey visual information to the CX (Scheffer et al. 2020; Hulse et al. 2021).

**Fig. 6:**
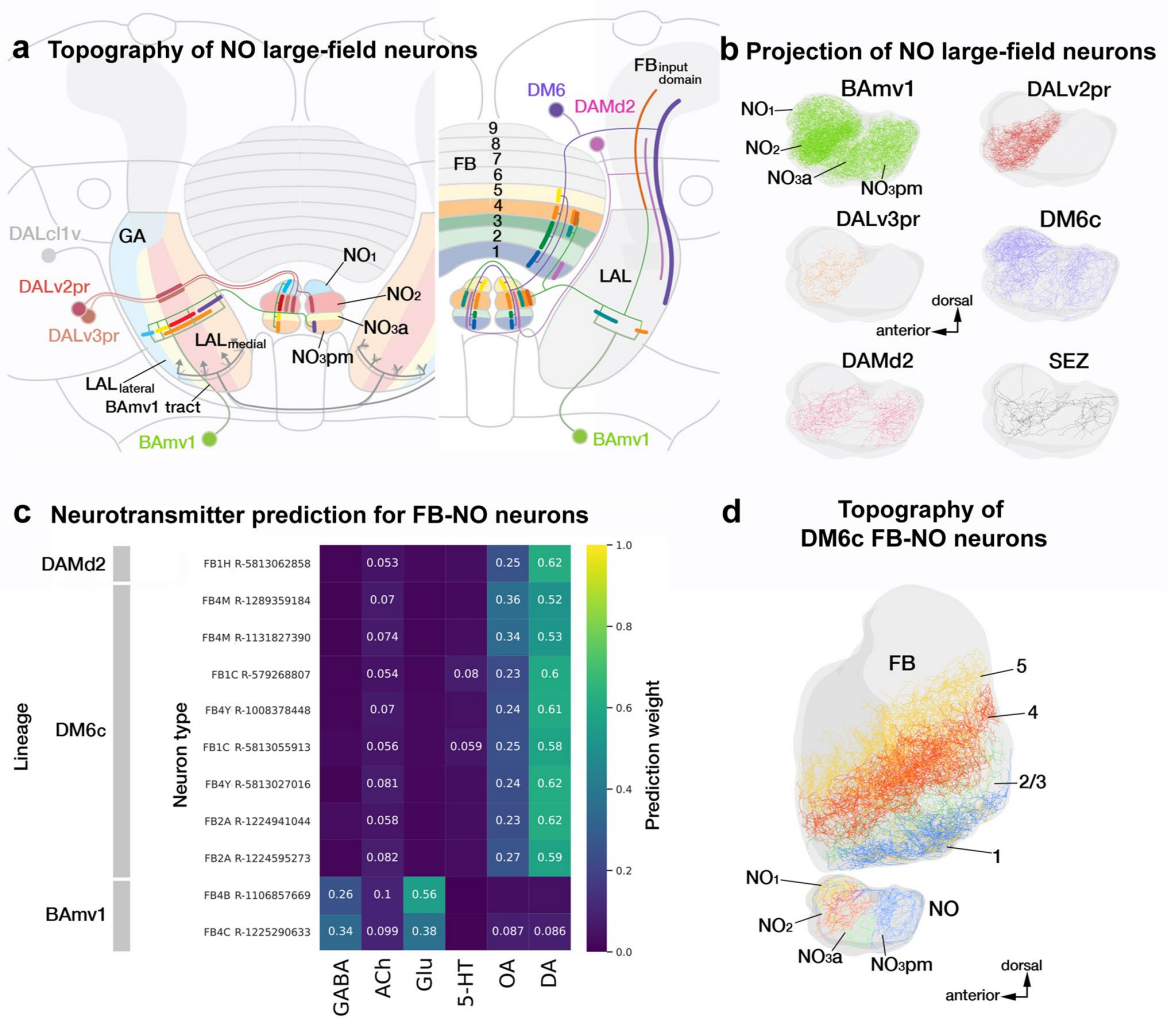
Organization of the noduli (NO) and the lateral accessory lobe (LAL). **a** Schematic frontal section of the brain hemisphere at the central level, visualizing the fan-shaped body (FB) and NO with surrounding neuropil compartments, as well as lineages with neurons that provide innervation of the NO. One major lineage, BAmv1 (light green), connects different vertical bands of the LAL with specific NO compartments. The position of the BAmv1 lineage-associated tract divides the LAL into a narrow lateral domain (cyan, yellow) and a medial domain (red, orange). As shown for FB large-field neurons, one detects a loose correlation between lateral-medial position of dendritic branches in the LAL and dorsal–ventral position of endings in the NO. Two neurons included in lineages DALv2pr and DALv3pr also form connections between LAL and NO. Right side of the panel (**a**) depicts the second major lineage, DM6, for NO input neurons. These neurons, in addition to the NO, have branches in the ventral half of the FB, as well as the FB input domain. Dorso-ventral position of the FB layer and dorso-ventral position of the connected NO compartment are strictly correlated, as indicated by corresponding colors. A small number of neurons of BAmv1 and DAMd2 also interconnect FB and NO in the manner indicated. **b** Plots of axonal arbors of NO-innervating neurons schematically shown in (**a**), presented in lateral view (anterior to the left, dorsal top). **c** Heatmap showing neurotransmitter predictions for different FB-NO neurons. **d** Plots of complete arbors of four representative DM6 FB-NO neurons in lateral view (anterior to the left, dorsal up), rendered in different colors. For abbreviations see Table 1

Resembling DM6c, the antero-dorsally located DAMd2 lineage (Supp. File 16) also contains neurons (FB1H, 1PBH) with shared arborization in the FB and NO (Fig. 5d; Fig. 6b, d). These neurons are also likely primary since they do not appear in the corresponding genetic clone. FB1H enters the dorsal SMP and gives off multiple dendritic branches pervading the CRE and SMP. The distal branch projects posteriorly, bypassing the EB laterally, to reach the ventral FB surface. The predominantly axonal tufts target the 1st layer of the FB and extend synaptic profiles well into the 4th layer (Fig. 5b; Supp. Figure 3a). Our neurotransmitter predictions suggest that these neurons are likely dopaminergic (Fig. 6c).

FB projecting components of DALcm1 (Supp. File 17) and BAmd1 (Supp. File 18) are quite difficult to resolve. Clones of the two lineages show that both tracts enter the neuropil in close proximity, and contain neurons that target the ventral (2nd) layer of the FB. The dense labeling of other members of this lineage precludes our ability to identify their dendritic domains to match and distinguish them in the hemibrain catalogue. In our tract map, we found these two bundles using the vertical lobe of the MB as a landmark. Owing to their similarity Scheffer et al. (2020) annotate the FB neurons that are part of these bundles as FB2B_a and FB2B_b—with one neuron per “type” falling into each bundle. These neurons, thus, might represent a similar circuit assembly motif as seen in the case of DALv2pr and DALv3pr. However, given their presence in the lineage clone, their developmental origins are likely not embryonic.

In the posterior cortex, lineages DM1 (Supp. File 19) and DM4 (Supp. File 8) each give rise to a pair of large-field FB neurons. DM4 contains FB1G and FB5AB (Fig. 5b), whose axons take a dorsal trajectory along the longitudinal superior medial fascicle (loSM) and extend multiple dorsally directed branches into the SMP and CRE. Fibers then make a U-turn around the EB and reach the FB from the anterior to target the 1–3rd and 5–6th layers, respectively. Sandwiched between these two neuron types, are the DM1-derived FB4K neurons. Their fibers curve ventrally around the posterior FB surface and fan out to innervate the middle layers of the FB (Fig. 5b). Extrinsic branches continue forward through the EB canal and branch bilaterally in the CRE.

#### Tiling the input domains: topographic mapping of the lateral neuropils onto the FB layers

As seen in the final leg of the anterior visual pathway, the DALv2-derived ER-neurons subdivide the bulb and the EB into anatomically identifiable compartments based on the developmental and functional properties of their inputs (Fig. 2a). We wondered whether similar topographical relationships also exist between the input and output domains of the FB neurons. The FB input domain (FB_ID_), unlike the minuscule BU, is represented by a large, longitudinally oriented neuropil domain that extends throughout the posteromedial SLP, SIP, SMP, CRE and then curves downward into the LAL (Fig. 5a).

The projection envelopes of DALcl2v, CP2d, BAmv1, and DM6 outside the FB span all aforementioned neuropil compartments. Neurons within each of these four lineages collectively innervate all layers of the FB. DPMpl2, on the other hand, projects to the dorsal layers of the FB, and remains dendritically restricted to the posterior part of the FB_ID_ (Fig. 5c). Further breakdown by FB layer reveals, within each lineage, a staggered organization of dendritic tufts that broadly maps the ventral layers of the FB to the antero-ventral part of the FB_ID_ and the dorsal layers to the postero-dorsal part of the FB_ID_ (Fig. 5c). Dendritic tufts of neurons innervating individual layers are organized in a successively tiling fashion with small overlapping edges. Although not as distinct as in the BU, this hints at an anatomical subdivision within the superior protocerebrum beyond the neuropil boundaries previously noted.

The most distinct mapping exists between the intermediate layers of the FB and the CRE. Neurons that innervate the 4th and 5th layers of the FB are the densest occupants of the CRE, wrapping entirely around the medial lobe of the MB (Fig. 5c). Neurons of layers 6–9 have progressively posteriorly located dendritic tufts, culminating in the small arbors of layer 9 neurons innervating the postero-dorsal part of the SLP/SIP. Layer 1 and 2 neurons have significantly broader dendritic arbors—collectively extending as far ventral as the tip of the WED (further details in the NO section).

Most neurons of the 1st FB layer form an exception to the topographical order followed by the other FB neurons. Layer 1 neurons have small dendritic tufts colocalizing with those of the layer 8/9 neurons (Fig. 5a, c). In conjunction with the BAmv1-derived atypical FB neurons that intrinsically interconnect the dorsal and ventral layers of the FB (as well as the asymmetrical body innervating SAF neurons; see AB section), the overlap of input domains of neurons constituting these regions suggests a structural and perhaps an important functional relationship between the dorsal and ventral extremes of the FB neuropil (Supp. Figure 3b).

#### Diversity of inputs to the FB large-field network

While five lineages form the numerical majority of the FB large-field network, only three of them account for about 74% of lateral inputs to this neuropil (Fig. 5d). Input channels constructed by the DALcl2v neurons are the most dominant (constituting ~ 28.6% of synaptic connections onto the FB large-field neurons). Within this population, the neuron type annotated as FB5V in the hemibrain, is the largest input channel (Fig. 5d). CP2d (23.2%) follows suit, as does BAmv1 (22.3%; Fig. 5d). Of the DM6 lineage, DM6c is the largest source of inputs to the FB (Fig. 5d). Interestingly, DPMpl2 falls shortly behind DM4d and only narrowly outnumbers the strength of inputs brought in by the minor lineage DAMd2. This is surprising, considering that synaptic input from the few FB neuron types formed by DM4d (2) and DAMd2 (1) comes close to, or even outranks that of the major lineage DPMpl2 (Fig. 5d). This pattern posits a more complex developmental organization of information flow into the FB than the numerical abundance would suggest.

Given the size of the FB_ID_, which covers a major portion of the superior protocerebrum and CRE, inputs to these FB neurons are extremely diverse, including more than 30 lineages across the entire brain. A large fraction of these neurons resides within the brain regions colloquially called “Terra Incognita”—highlighting the difficulty in understanding the role of the FB inputs in shaping navigational behaviors. Amid this diversity, a few prominent connections stand out. We focused on a dedicated set of inputs to the FB large-field neurons from the higher-order olfactory structure, the mushroom body (MB). The MB output neurons (MBONs) belong to twelve lineages, seven of which strongly output onto the FB large-field neurons (Fig. 5e). These synaptic contacts are predominantly located in the CRE, and thus the MB→FB networks preferentially target the 3–6th layers of the FB (Hulse et al. 2021).

Along with being the numerically largest lineage, DALcl2v also has the most developmentally diverse set of MBON inputs (CP2d, DALcm1m, DALcm1v, and DALv2). BAmv1 also displays such input diversity, but is more selective: its strongest upstream MBON partners belong to the DAMd1/SMPad3 lineage (Fig. 5e). The CP2d and DM4d FB neurons display stark developmental selectivity, receiving MBON inputs almost exclusively from DALv2 and BAmas1/FLAa2, respectively (Fig. 5e). Interconnecting the flange (FLA), MB, and the FB, the BAmas1→DM4d network represents a likely candidate for a pathway tying spatial location to learnt feeding behaviors. MBON inputs to the other FB lineages are quite weak (Fig. 5e).

Contrary to the strict DALcl1/2d-TuBu→DALv2-ER ordering of the AVP, we do not observe any clear lineage-based organizational principles of inputs to FB neurons. The spatial tiling of input and output domains of these neurons themselves, irrespective of developmental origin, seems to be a more significant aspect of the FB network. Given the virtual absence of recordings from these neurons, it is not yet possible to enunciate any substantive conclusions as to how the structural/developmental properties of inputs to this neuropil relate to their functions.

### The paired noduli: a second entryway of topographically ordered, multimodal sensory input into the CX

Located ventrally of the fan-shaped body (FB) and ellipsoid body (EB) is the bilateral paired globular structure called the noduli (NO; Fig. 6). In *Drosophila*, the NO are composed of three compartments arranged much like the bow of a ship: the dorsal (NO_1_), the intermediate/medial (NO_2_), and the posterior/ventral (NO_3_) compartments. NO_2_ and NO_3_ can be further subdivided into two and three sub-compartments, respectively (Wolff et al. 2015). These compartments and sub-compartments represent serial homologs of FB layers or EB annuli/domains in the NO. However, unlike the FB and EB, the noduli does not display compartmentalization along the transverse axis—i.e., they appear to lack a columnar organization (Hanesch et al. 1989; Lin et al. 2014; Wolff et al. 2015; Wolff and Rubin 2018). Thus, each NO compartment is innervated by converging collaterals of small-field neurons that connect the protocerebral bridge (PB), FB, and EB with the NO, originating from all four CX DM lineages of the contralateral hemisphere. This compacted structure, lacking complex intrinsic circuitry, thus forms a dedicated input neuropil broadly influencing the spatial representation in the CX (Hulse et al. 2021).

Neurons providing inputs from lateral neuropils to the NO fall into two broad groups. The first connects the lateral accessory lobe (LAL) directly to the NO (collectively called LAL-NO neurons in the following). The second, as described above, arborize in the NO as an extension of their FB innervation (FB-NO neurons; Fig. 6a).

LAL-NO neurons include 9 neurons per hemisphere, which, using our tract map, we were able to assign to BAmv1 (neurons GLNO, LNOa, LCNOp, and LCNOpm; 6PBH; Supp. File 3) as well as the primary components of the DALv2 (DALv2pr; neuron LNO1; 2PBH; Supp. File 14) and DALv3 (DALv3pr; neuron LNO3; 1 PBH; Supp. File 15) (Fig. 6a; Hartenstein and Kandimalla, in preparation). Consistent with our assignments, a recent survey of the developmental fate of primary neurons, using genetically immortalized neuronal labeling, confirmed the embryonic origins of LNO1 and putatively LNO3 (Truman et al. 2022). Both LNO1 and LNO3 neurons selectively innervate NO_2_, with LNO1 terminals occupying the ipsilateral hemisphere and LNO3 extending bilateral arbors most dominant in the contralateral hemisphere (Fig. 6). BAmv1 neurons innervate all NO compartments on the ipsilateral hemisphere, with the exception of one neuron innervating the posterior sub-compartment of NO_3_ (NO_3_p; neuron LCNOp) in the contralateral hemisphere.

LAL-NO neurons create a systematic mapping between the LAL and the NO compartments. In the adult brain, the BAmv1 tract, while passing through the LAL, delineates two distinct domains—a medial and lateral domain (Fig. 6a). Each of these domains is further divided, by the location of the primary branch point and the dominant projection pattern of the proximal dendritic tuft of the different LAL-NO neurons, into two vertical bands (Fig. 6a). Neuronal arbors in the LAL_lateral_ tend to be narrow and elongated while those in the LAL_medial_ are more dispersed. Broadly, from lateral to medial, the four bands map onto the dorso–ventral axis of the NO (color-coded in Fig. 6a). For instance, the most lateral band of the LAL, which also includes the gall (GA), is connected to the NO_1_ by the BAmv1-derived GLNO neurons (LAL.s-GAi.s-NO_1_i.b in Wolff and Rubin (2018)). At the other end, the most medial LAL band maps onto the postero-ventral NO sub-compartments, NO_3_m and NO_3_p (Fig. 6a). The dendritic arbors of the contralaterally projecting BAmv1 neuron (LCNOp) also occupy the most medial band of the LAL (Fig. 6a). This spatial ordering of input and output domains of the LAL-NO neuron population suggests potential functional segregation of inputs and feature selectivity of the constituent neurons.

Recent functional recordings of a subset of LAL-NO neurons have indeed revealed differences in their response properties (Currier et al. 2020; Lu et al. 2022; Lyu et al. 2022). Arborizing in the LAL_medial_, the LNO1 (DALv2pr) and LNO2 (BAmv1) neurons both innervate the NO_2_ and respond to optic flow stimuli. However, these neurons with differing developmental origins exhibit differential direction-tuning and downstream partner selectivity. Whereas LNO1 neurons preferentially respond to progressive optic flow, LNO2 responds to regressive visual motion (Lu et al. 2022; Lyu et al. 2022). Both neuron types are also weakly activated by self-movement in the corresponding directions in the absence of visual cues (Lu et al. 2022). LNO1 and LNO2 neurons predominantly target and inhibit the PFNv and PFNd CX small-field neurons, respectively (Lu et al. 2022; Lyu et al. 2022). This feature and partner selectivity is an essential part of the LNO1, LNO2, PFNd, and PFNv network in building the allocentric traveling representation in the CX (Lu et al. 2022; Lyu et al. 2022).

Functional studies of the NO_3_a innervating LNOa (BAmv1) neuron, forming dendritic arbors in the LAL_lateral_, also show properties that are distinctively different from the LAL_medial_ neurons. Dendrites of LAL_lateral_ neurons largely overlap with those of the ER1 neurons. In line with this anatomy, the LNOa neurons, like ER1, have also been shown to respond to mechanosensory stimuli associated with wind-induced arista movements (Currier et al. 2020). Connectivity partner analysis also suggests that the other LAL_lateral_ population, GLNO, might encode mechanosensory information (Hulse et al. 2021). These observations suggest that the LAL might be divided into two functional domains—with synaptic profiles in the LAL_lateral_ being putatively mechanosensory and LAL_medial_ processing visual or other sensory or valence modalities (e.g., olfactory information via MB output neurons; Hulse et al. (2021)). This spatially structured synaptic connectivity creates a functional map between the LAL and the NO sub-compartments (Fig. 6a).

A topographic ordering is also evident among part of the neurons that share FB and NO innervation (Fig. 6a, d). The 8 FB-NO neurons derived from lineage DM6c, project to individual layers forming the ventral half of the FB. Neurons focusing on the most ventral FB layer 1 project to the postero-ventral sub-compartments NO_3_p/m, while neurons with further dorsal FB innervation reach further antero-dorsal NO compartments (Fig. 6a, d). While the FB arbors of these neurons are not strictly limited to individual FB layers, their NO arborization is confined to specific compartments. The NO innervation is bilateral, covering corresponding sub-compartments on both sides (Fig. 6a, d). Aside from DM6, BAmv1 and DAMd2 also generate a small number of FB-NO neurons. These roughly match the topography established by DM6c neurons: the three BAmv1 FB-NO neurons innervate the 3rd and 4th layers of the FB and reach NO_2_; DAMd2 innervates layer 1 and projects to NO_2_/NO_3_ (Fig. 6a). Interestingly, the dendritic arbors of FB-NO neurons formed in the FB_ID_ are far broader than those of the large-field neurons that innervate the FB only—spanning throughout the SMP, CRE, as well as the LAL with weaker protrusions into the SLP (Fig. 6a).

The functional properties of FB-NO neurons are largely unexplored. However, EM neurotransmitter predictions (Eckstein et al. 2020) suggest an organizational principle akin to the protocerebral bridge. Corroborating their assignment to DM6c, the synaptic profiles of the FB-NO neurons in this tract are indeed strongly predicted to be dopaminergic (PPM3 cluster; Fig. 6c). The individual DAMd2 neuron FB1H is also likely dopaminergic (Fig. 6c). Along with the OA-VPM3 octopaminergic neurons, the DM6c and DAMd2 neurons are a likely source of neuromodulation of activity profiles across the FB and the NO based on the behavioral or sensory state of the animal. Given their interaction with the LAL-NO neurons, this population might also be required for motion-dependent synaptic plasticity in the FB. Finally, the two BAmv1 neurons are likely inhibitory, with the neurotransmitter predictions only slightly favoring glutamate over GABA (Fig. 6c).

### BAmv1: the building block of the asymmetrical central complex structure

The only clearly asymmetric structure in the *Drosophila* brain is the aptly named asymmetrical body (AB). Located along the ventral surface of the fan-shaped body (FB), the AB is a paired structure with the right compartment being consistently bigger than its left counterpart (Pascual et al. 2004; Wolff et al. 2015). About 7–10% of flies display an interesting symmetry in this structure, which has been speculated to be associated with poorer memory performance (Pascual et al. 2004). Despite being embedded amid the teeth of the FB, neuronal markers do not specify any clear columnar organization of the AB neuropil. However, small-field columnar neurons innervating the AB(Right) tend to be biased to the medial or lateral edges in correspondence with the columns they occupy in the FB (Supp. Figure 5). Owing to its significantly smaller size, innervation biases, although present, are not as evident in the AB(Left) (Supp. Figure 5).

**Fig. 7:**
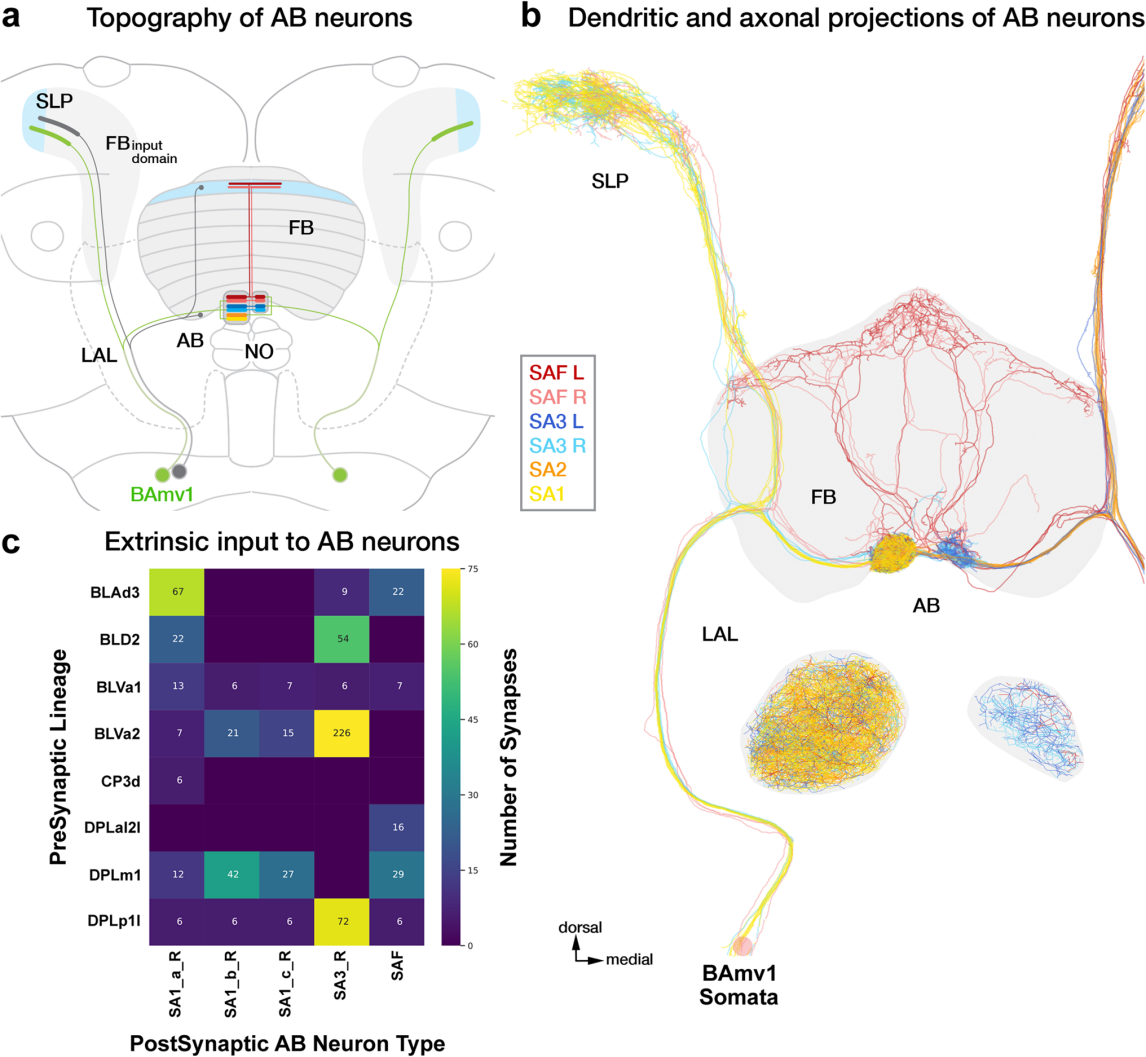
BAmv1 builds the asymmetrical brain structure along the ventral surface of the fan-shaped body (FB). **a** Schematic frontal section of the brain at a central level, visualizing the FB and asymmetrical body (AB) with surrounding neuropil compartments. Input to the AB is exclusively provided by 16 pairs of BAmv1 neurons, forming the classes SA1, SA2, SA3 and SAF. Dendritic input of all of these neurons is derived bilaterally from the posterior part of the FB input domain; axonal output reaches preferentially the right AB, as visualized in the diagram. Note additional projection of neuron class SAF to layers 1 and 8 (highlighted in blue) of FB. A similar bifurcated projection is seen in atypical BAmv1 neurons FB1I and FB1J (schematized in grey). Excluded in this schematic is the sparse arborization of the DM4-FB1G neuron in the AB(Right). **b** Rendering of the hemibrain BAmv1 AB neurons, color coded as in schematic (**a**). Inset shows AB at higher magnification. **c** Heatmap depicting input neurons and their lineages of origin (vertical axis) on different classes of AB neurons (horizontal axis). Note that only a few lineages provide the large majority of input; each AB neuron group is targeted by a distinct combination of input lineages. For abbreviations see Table 1

Inputs to the AB are entirely formed by the BAmv1 lineage (Fig. 7; except for sparse innervation of the DM4 FB1G neurons, see Supp. File. 8). AB neurons resemble the BAmv1 large-field neurons of the FB in their trajectory, primary branching location, and entry portal into the CX (Fig. 7a, b). The proximal dendritic arbors of all AB neurons extend dorsally and arborize in the SLP. Individual neuron types are characterized by the innervation pattern of their distal axonal tufts in the AB compartments (Wolff and Rubin 2018). The most prominently asymmetric neurons exclusively target the AB(Right) irrespective of their hemisphere of origin. The tracts and the innervation of the right BAmv1 neurons with this morphology annotated as SA1 in the hemibrain (SLP.s-ABi.b in Wolff and Rubin (2018)), remain restricted to the ipsilateral hemisphere. The corresponding BAmv1 neurons from the left hemisphere, the hemibrain SA2 type (SLP.s-ABc.b in Wolff and Rubin (2018)) cross the midline antero-ventrally of the AB(Left) and arborize in the AB(Right). The remainder of the AB neuron types (SA3, SAF) originate from both hemispheres and target both AB compartments, although they are denser in the AB(Right) than the AB(Left). The SA3 neurons (SLP.s-ABic.b; Wolff and Rubin (2018)) extend a set of tufts into both compartments, which are interconnected by the neuron tract crossing over into the contralateral hemisphere. SAF neurons (SLP.s-ABic.b-FB*l*8.b in Wolff and Rubin (2018)) in addition to bilateral AB innervation also extends projections dorsally along the anterior surface of the FB into the 8th layer (Fig. 7).

The localization of the dendritic tufts of the BAmv1 AB neurons in the dorsal SLP is akin to those of the dorsal FB large-field neurons (see Fig. 5c). Furthermore, the interconnection of the (ventrally located) AB and the (dorsal) 8th layer of the FB by the SAF neurons is reminiscent of the FB1I and FB1J BAmv1 neurons which form a similar motif with the 1st and 8th layer (Fig. 7a, b; Supp. Figure 3b). The same dorsal–ventral link is also established by the FB intrinsic vDeltaA pontine neurons (Supp. Figure 5). This innervation pattern by three different groups of neurons supports the hypothesis that a functional relationship exists between the two extremes of the FB, and that the AB lends an asymmetry to this circuit.

Inputs from lateral neuropils onto the AB neurons of the right BAmv1 lineage are developmentally heterogenous (Fig. 7c). They belong to eight lineages, although only five display strong synaptic connections—BLAd3/SLPal3, BLD2/SLPav2, BLVa2/LHa3, DPLm1/SLPpm1, and DPLp1/LHp2 (Fig. 7c). Each AB neuron type receives inputs from a discrete combination of these lineages. SA1_a neurons receive the strongest inputs from BLAd3 neurons (Fig. 7c). The two other subtypes of SA1, SA1_b and SA1_c, share strong inputs from DPLm1 and BLVa2. The bilaterally projecting SA3 (right) neurons also receive strong inputs from BLVa2 (Fig. 7c). This connectivity strength outranks any other lateral input strength to the BAmv1 AB neurons—almost 10- and 15-fold higher than their outputs to SA1_b and SA1_c neurons, respectively (Fig. 7c). SA3 neurons are also targeted by DPLp1 and BLD2 neurons. Inputs to SAF neurons are weaker than the other major connections surveyed, and belong to DPLm1, BLAd3, and DPLal2 (Fig. 7c). The convergence of neurons from a few lineages onto the classes of AB neurons (BAmv1) resembles the connectivity motif displayed by DALcl1d and DALcl2d convergence onto ER-neurons (DALv2).

Due to the restricted nature of the hemibrain imaging volume, the dendritic tufts of the BAmv1 AB neurons of the left hemisphere are missing. This precluded any analysis of differences in inputs across the hemispheres.

The functional relevance of the AB still largely remains a mystery. Pascual et al. (2004) first correlated weaker long-term memory performance in odor-conditioning paradigms with symmetrical AB morphology. A more recent study has shown Ca^2+^ oscillations in the vDeltaA neurons of the AB correlated with the nutritional state of the animal (Musso et al. 2021). This activity can be influenced by the manipulation of the BAmv1 SA1/SA2 neurons, as is expected based on their strong connectivity (Musso et al. 2021). Despite these observations, no conclusive evidence points to the role of the asymmetry of this structure.

### Conserved organizational principles of the central complex inputs across taxa

Despite a wide degree of divergence across phylogenetic time and disparate ethological and navigational strategies, neuropil compartments of the central complex (CX) display a remarkable degree of conservation across insects (Strausfeld 1976; Honkanen et al. 2019). The volume of neuropil compartments, including those comprising the CX, are formed by the branches and synaptic contacts of identifiable neurons that emerge from fixed lineages (Ito et al. 2013; Yu et al. 2013; Lovick et al. 2013; Wong et al. 2013). Indeed, the conservation of embryonic neuroblasts in different insects has been documented for a few species, and the axon tracts and fascicles in the adult (which reflect the mature lineages that comprise them) are comparable and presumed homologous between diverse insect clades (Zacharias et al. 1993; Biffar and Stollewerk 2014; Farnworth et al. 2020). Within lineages, as affirmed by several studies in *Drosophila* (Lee 2017; Sullivan et al. 2019; Mark et al. 2021), a given neuroblast will deploy temporal and hemilineage mechanisms during its proliferation to produce diverse classes of neurons, which can now be assessed beyond morphology with the advent of connectivity information afforded by the connectome.

The focus of this study is the large-field elements that provide input from various brain regions to different CX compartments and process/reformat information within the CX. An important aspect of this work beyond elucidating the developmental organization of CX input channels at the lineage→neuron type→circuit levels in the fly connectome, is to facilitate comparative analyses which may yield general insight on the evolution of insect nervous systems. Although there are 199 unique large-field neuron types innervating the CX, they all derive from a small number (14) of lineages (*Drosophila*). Granted, precise homology between specific large-field elements in different insects is difficult to assign without access to specific molecular markers or synaptic connectivity data. However, the core circuit architecture of highly regular small- and large-field elements that project along stereotyped tracts and form CX columns and layers, respectively, can be homologized with individual neurons described in other insects at the lineage level based on their relative cell body locations, tract trajectories, and innervated compartments (taking anatomical distortions of different insect brain morphologies into account).

**Fig. 8:**
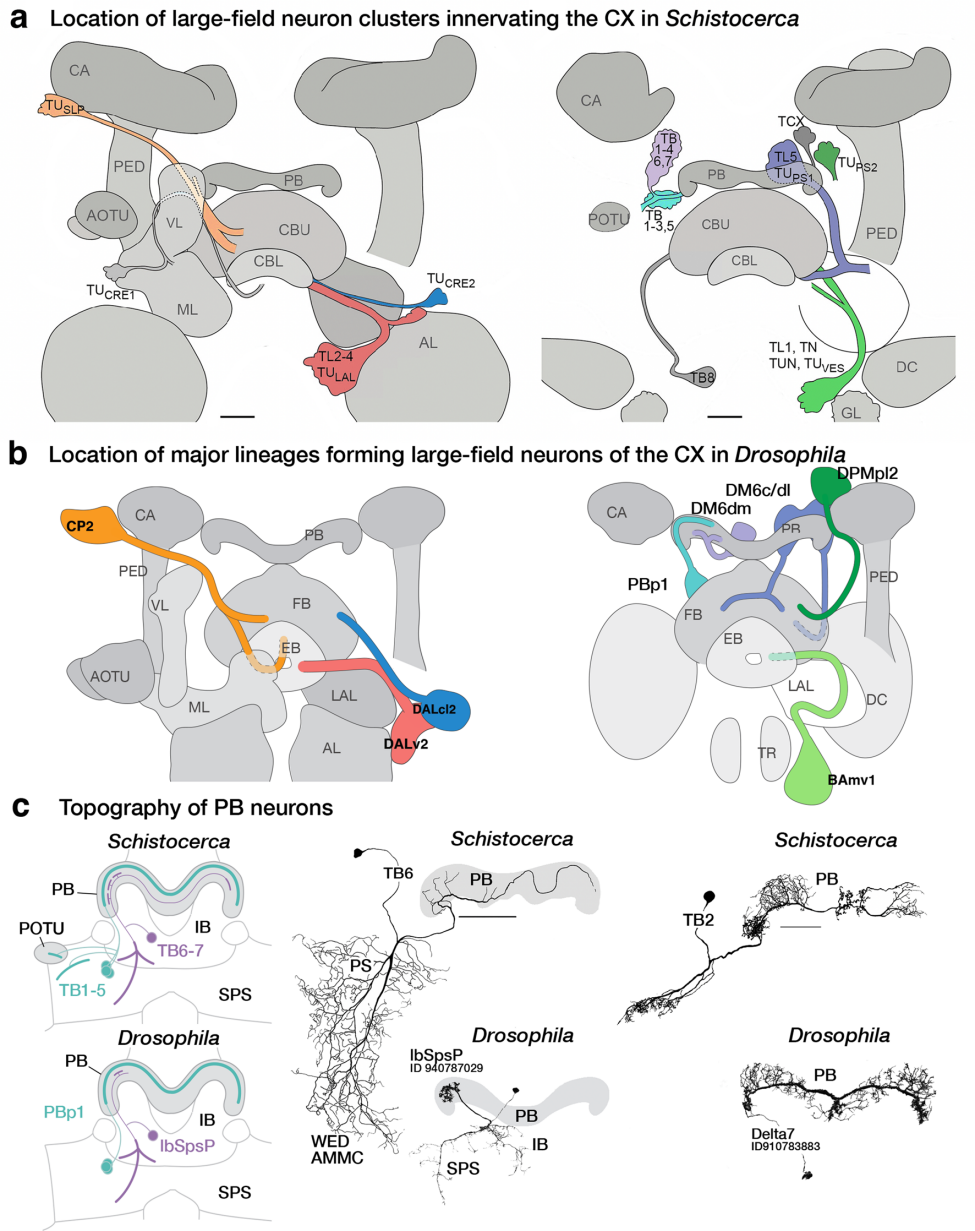
Topography of major clusters of CX large-field neurons in two insects, *Schistocerca* and *Drosophila*. **a**, **b** Schematic anterior view of major groups of CX large-field neurons (color coded) in relationship to central brain compartments (gray) in *Schistocerca* (**a**) and *Drosophila* (**b**). Left panels depict groups of neurons with cell bodies in the anterior brain; right panels are those with cell bodies in the posterior brain. Graphics and nomenclature for *Schistocerca* are adapted from (von Hadeln et al. 2020) (with permission). **c** Comparison of the architecture of the protocerebral bridge (PB) in *Schistocerca* (upper panels) and *Drosophila* (lower panels). The left panels schematically compare neurons of PBp1 (*Schistocerca*: TB1-5) and IbSpsP (*Schistocerca*: TB6, TB7). Note the absence of extra-PB dendritic branches (POTU) of PBp1 in *Drosophila*. Middle and right panels show line drawings (top, for *Schistocerca*) and digital plots from hemibrain (bottom, for *Drosophila*) of homologous neurons TB6/IbSpsP and TB2/Delta7, respectively. For abbreviations see Table 1

In the following section, we survey the evolutionarily conserved large-field elements in the CX as it reflects a homologous lineage ground plan across insect taxa. The locust (*Schistocerca gregaria*) will be used as the primary basis for comparison as its large-field CX network is the most comprehensively described and is generally representative of what is observed in other insects. Despite its divergence 300 + million years ago, hemimetabolous lifecycle, and differences in brain size/shape, compelling homologies of individual neurons to the fly lineages can be made based on the aforementioned criteria (Fig. 8).

#### Large-field inputs to the central body lower division: ER- and ExR neurons

Of the neuron classes which form large-field elements in the *Drosophila* CX, the ER-neurons, which are derived from the DALv2 lineage (Hanesch et al. 1989; Wong et al. 2013; Omoto et al. 2018), are the best understood. In other insects, all large-field neurons which innervate the homologous structure to the ellipsoid body (central body lower division; CBL) are designated as TL neurons (tangential neurons of the CBL), regardless of their developmental origin. Indeed, some TL neurons have cell bodies located in other spatial positions within the cell body rind (and thus derive from other, non-DALv2 lineages). These non-DALv2 TL neurons are homologous to the *Drosophila* extrinsic ring neurons (ExR), distinguished due to their broader arborization envelopes and distinct neuroblast lineage origins (see below).

TL2-TL4, and the recently described TL7, are homologous to DALv2 ER-neurons (von Hadeln et al. 2020; Hensgen et al. 2021b). In the locust, their cell bodies are clustered dorso-medial of the antennal lobes. TL2-4 project postero-laterally around the posterior surface of the AL and then turn medially to reach the EB via one of the isthmus tracts (isthmus tract 2), a complex fiber bundle that corresponds to the *Drosophila* LEa/p tract (Fig. 8a). TL2-TL3 protrude into the bulb which is split into medial and lateral domains, forming large microglomerular synaptic complexes with upstream neurons that connect the AOTU lower unit to the medial/lateral bulbs via the tubercle-accessory lobe tract (Träger et al. 2008). These upstream neurons, called TuLAL neurons, have cell bodies clustered dorso-laterally of the antennal lobe and are homologous to TuBu neurons—thus likely derive from the homologous lineages DALcl1/2. Although taking a seemingly tortuous trajectory in comparison to the *Drosophila* TuBu tract, the tubercle-accessory lobe tract formed by TuLAL neurons conforms to the constraints of a much larger and tilted MB and a more ventrally located lateral complex. In both *Drosophila* and locust (and all other insects examined), the tract runs medially “behind” the alpha/alpha’ lobe (posteriorly in *Drosophila*, dorsally in locust). TuLAL neurons receive input from the visual system completing a circuit called the sky-compass pathway, named for its role in transmitting navigationally-relevant skylight visual cues to the central complex (Heinze and Homberg 2007). In *Drosophila*, the homologous circuit was called the anterior visual pathway (AVP), using an anatomy-based nomenclature as to remain agnostic on the nature of the visual cues transmitted (Omoto et al. 2017). Large-field neurons that connect the bulb to CBL (DALv2) as well as their upstream partners (DALcl1/2), with similar cell body positions and axon trajectories, have also been described in dung beetles (Coleoptera) (el Jundi et al. 2014, 2015), butterflies (Lepidoptera) (Heinze et al. 2013), and honeybees (Hymenoptera) (Hensgen et al. 2021a), among other species. This observation speaks to the deep homology of this lineage set to form the visual input pathway to the CX. Differences in wiring features of individual elements between species may reflect neuroethological adaptations. For example, TuBu→ER convergent connections in *Drosophila* are formed by single microglomerular complexes, whereas in locust and butterflies, individual TL and TuLAL neurons display multiple synaptic specializations in the bulb. Although the functional consequence of this difference remains to be seen, we suspect that it reflects the visual ecology of the animal, and may manifest as modifications to convergence/divergence, conjunctive coding of visual features, and constraining or expansion of receptive fields.

The locust TL4 neuron somata cluster together with TL2/3 and extend along the same tract, but rather than innervating the bulb, project fibrous arbors ventrally into the LAL. These neurons, which have also been observed in butterflies (Heinze et al. 2013) and are likely present in other insects as well, are proposed homologs of DALv2-ER1 in *Drosophila*. Owing to this similarity, we suspect that TL4 neurons across species convey information regarding directional airflow/antennal movements to the compass system (Okubo et al. 2020).

The locust TL5 is distinct from TL2-TL4 in that its cell body is located postero-medially adjacent to the PB, indicating a different neuroblast origin. This neuron, the likely homolog of which has also been described in honeybees (*Apis mellifera*) (Hensgen et al. 2021a), is presumed dopaminergic and is likely homologous to the DM6 lineage-derived PPM3-DAN ExR2 in *Drosophila*. Somata of the TL5 cluster are located ventro-posteriorly of the PB, flanking the posterior slope (PS). Fibers continue forward along the w-bundle, which is part of the MEF bundle that carries the *Drosophila* DM6 neurons. Interestingly, this DM6-derived dopaminergic neuron exhibits drastic differences in neuropil compartment innervation across taxa. The locust TL5 arborizes within the PB and continues anteriorly before broadly innervating the lateral complex and CBL (von Hadeln et al. 2020), whereas the honeybee TL5 bypasses the PB and innervates the LAL and CBL (Hensgen et al. 2021a). The *Drosophila* ExR2 also bypasses the PB and innervates the EB and the lateral complex bilaterally (Omoto et al. 2018). If these neurons are indeed homologous, the differences in wiring properties might hint at a general evolutionary-developmental principle: neuromodulatory circuit elements—which do not convey labeled line information, rather modulate information flow in entire networks—are awarded a higher degree of flexibility.

The TL6 neuron described in honeybee (*Apis mellifera*) is most likely homologous to ExR8 (or possibly, ExR7), which, in *Drosophila*, form part of the DM3/DM4 lineages (atypical developmental profiles described in the ExR section). Like *Drosophila* DM4-ExR8, honeybee TL6 somata are located close to TL5 (i.e., lineage DM6) and project along the ventral border of the PB (the location of the MEF, used by DM4 neurons). At the level of the FB the fiber splits into an upper axonal branch innervating the CBL/EB, and a lower dendritic branch which broadly arborizes in the PS (Hensgen et al. 2021a). Unlike *Drosophila* ExR8 neurons, TL6 lacks arborzation in the NO and only extends a small sliver of branches ventrally from the CBL/EB in the contralateral hemisphere.

An additional TL neuron bearing strong homology to the *Drosophila* ExR7 neurons has been identified in the Bogong moth (*Agrotis infusa*) by de Vries et al. (2017). Referred to as TL(GA-BU-POTU), this neuron is part of the DM3 lineage and closely resembles the CBL/EB innervation as well as the symmetric bilateral projections into the lateral complex displayed by the ExR7 neuron. Its key differentiating feature is a pair of proximal branches that it extends laterally just as the DM3 tract enters the neuropil. These branches proceed to densely arborize in the posterior optic tubercle (POTU), a small neuropil compartment ventro-lateral to the PB that is apparently absent in *Drosophila* (see comparative PB section for detailed description), and sparsely in the surrounding PS (de Vries et al. 2017). The POTU innervation of the TL(GA-BU-POTU) neuron suggests that its function might depend on the integration of circadian signals—a class of computations that are required for the Bogong moths to perform their iconic migratory behavior (Heinze and Warrant 2016; Warrant et al. 2016). The absence of this neuropil, and the ExR7 branches within, are in line with the absence of time-compensated migratory behaviors in *Drosophila*.

Lastly, TL1 is also a non-DALv2 derivative. Its cell body is located ventro-posteriorly of the antennal lobe and extends a fiber dorsally behind the posterior face of the AL, projects a diffuse proximal neurite in the LAL, and enters the CBL on its dorso-posterior face through isthmus tract 1. This neuron is highly recognizable across species and has been described in honeybees and butterflies (Heinze et al. 2013; Hensgen et al. 2021a). In *Drosophila*, the BAmv1-derived ExR4/ExR6 neurons are the likely homologs. In addition to ExR4/ExR6 of the EB, BAmv1 gives rise to multiple large-field elements which follow the same tract but innervate different compartments of the CX (Fig. 1g). In the locust, other neurons (TU_VES_, see below) cluster around TL1 neurons and display the same projection pattern—strengthening the homology argument for the BAmv1 lineage across species. In general, BAmv1 neurons appear highly stereotyped between species, suggesting that they fulfill a fundamental role and their evo-devo→wiring properties are preferentially left unmodified.

#### Large-field neurons of the protocerebral bridge

In *Drosophila*, large-field neurons (atypical) of the protocerebral bridge derive from PBp1 and DM6dm (along with few neuromodulatory neurons of unknown origins; Fig. 4a, b). In other insects, tangential neurons of the PB are called TB neurons (TB1-8), and also belong to two tracts possibly homologous to those observed in *Drosophila*. The large-field PB neurons which have received the most attention in *Drosophila* are the PBp1-derived intrinsic neurons, Delta7. The connectivity profiles of Delta7 over the width of the PB have led to their proposed role in stabilizing and reformatting the profile of the activity “bumps” in the small-field networks (Turner-Evans et al. 2020; Lu et al. 2022; Lyu et al. 2022). The locust homologs of the Delta7 are TB1 and TB2, which, similarly to PBp1-derived neurons in flies, exhibit coarsely distributed cell bodies lateral of the PB (seated more dorsally due to the tilt in the neuraxis). They enter the lateral extent of the PB via a bundle of fasciculated axons. There are two clear differences in the distribution of input–output synapses between *Drosophila* and the locust: (1) fly Delta7 neurons have spatially confined outputs in specific glomeruli and broad inputs across the rest of the PB, while locust TB1/2 exhibit similar confinement of both inputs and outputs; (2) fly Delta7 neurons are intrinsic to the PB, whereas locust TB1/2 display additional input neurites in the POTU. Computational modeling suggests that the functional consequence of such a glomerulus confinement difference manifests itself in the dynamics of the simulated bump—the fly bump can change its angular phase more rapidly than the locust bump, consistent with the maneuverability of flight behavior between the two species (Pisokas et al. 2020).

The dendrites of locust TB neurons in the POTU receive significant input from the accessory medulla, directly from the circadian clock via pigment-dispersing factor (PDF) neurons (Held et al. 2020). The implementation of time compensation in navigational behaviors (the ability to maintain a global spatial reference frame by adjusting for the apparent motion of celestial cues by integrating their movement over time) requires input from an internal clock, and the POTU pathway is postulated as its neural substrate. Interestingly, the POTU pathway has been predominantly described in insects which would rely on time compensation, such as migratory moths, locusts, and butterflies (Heinze et al. 2013; Beetz et al. 2015; Heinze and Warrant 2016; Warrant et al. 2016), or central place foragers such as honeybees that use celestial cues to find food at different times of day (Kaiser et al. 2022). It is not apparent in dung beetles (*Scarabaeus*) and flies, which likely use a snapshot, or fixed memory, mechanism to navigate (el Jundi et al. 2014, 2015; Giraldo et al. 2018). Therefore, the wiring differences of these homologous neurons appear to reflect the ethological demands of each species.

In *Drosophila*, other PBp1 derivatives include the SpsP and P6-8P9 neurons. Given the arborization of SpsP in the posterior slope, the candidates for its homologs in the locust are TB4 and TB5. P6-8P9 is likely homologous to TB3—neurons from both species display a concentration of varicose arborizations in sparse glomeruli and smooth dendritic arbors across one hemisphere of the PB (von Hadeln et al. 2020). As with Delta7-TB1/2 neurons, the SpsP and P6-8P9 neurons also exhibit species-specific differences. TB3 has dendritic branches in the POTU (which is absent in *Drosophila*), and TB4/5 innervate the entire width of the PB. These morphological differences further allude to the evolutionary flexibility of PBp1.

The remaining locust PB large-field neurons include TB6, TB7, and TB8 (von Hadeln et al. 2020). TB6 and TB7 are likely homologs of DM6dm-derived IbSpsP neurons (Fig. 4b). The *Drosophila* IbSpsP somata cluster is located postero-medially of the PB and sends its axon tract ventrally. These tracts subsequently bifurcate, sending a ventrally projecting dendritic arbor into the PS, and a dorsally projecting axon that enters the PB at a position medially of the PBp1 neurons. Locust TB6 and TB7 somata are clustered dorso-laterally of the PB, a difference again likely attributable to a difference in neuraxis tilt. TB6/7 and IbSpsP neurons bear striking resemblance in the trident-like splay of their ventrally projecting dendritic ramifications. Across species, this neuron population displays a “pseudo-columnar” morphology, with the locust TB6/7 PB innervation spanning a wider domain of the PB than those of the *Drosophila* IbSpsP neurons. The functional consequence of this difference remains unknown.

TB8 somata are located in the anterior cortex, ventro-medially of the antennal lobes (AL) (von Hadeln et al. 2020). Their fibers project posterior and extend proximal branches laterally into the WED, LAL, and PS before further extending towards the optic lobes (Homberg et al. 2013). The distal branch continues further posteriorly before extending dorsally, bifurcating, and innervating the PB. These neurons have been shown to be octopaminergic (part of the OA1/TA cluster), and have historically also been referred to as O2 neurons (Homberg et al. 2013). The TB8/O2 axon trajectory, cell body location, and neurotransmitter posit it as the counterpart of the *Drosophila* OA-AL2i1 neurons (Fig. 4b) (Busch et al. 2009; Homberg et al. 2013; Wolff and Rubin 2018). Beyond neuron matching, this homology allows us to ascribe the OA-AL2 octopaminergic neuron cluster in *Drosophila*, neurons within which do not apparently appear in any neuroblast lineage clone, to the OA1/TA cluster in the locust brain (Kononenko et al. 2009; Busch et al. 2009).

Finally, the homologs of the *Drosophila* LPsP neurons are not evident in the locust catalog generated by von Hadeln et al. (2020), and are suspected to be absent (Timm et al. 2021). A neuron type with a similar cell body location, near the esophageal foramen, and the likely source of dopaminergic input to the PB, has been observed in another dipteran (blowfly—*Calliphora erythrocephala*) (Nässel and Elekes 1992; Timm et al. 2021). This neuron was designated as the constituent of the T1 cluster, a nomenclature and homology which was later carried forward by Mao and Davis (2009) in *Drosophila*. Based on a broad survey of tyrosine hydroxylase immunostaining across taxa, Timm et al. (2021) suspect that this T1/LPsP neuron is specific to Diptera. However, despite the incomplete dye-fill, a neuron with a strikingly similar tract and branching morphology, the TB-avm-1 neuron, has been identified in butterflies (Heinze et al. 2013). While its neurotransmitter identity has yet to be ascertained, we suspect that it represents the butterfly homolog of the *Drosophila* LPsP neuron. As among the TL neurons, it appears that the neuromodulatory circuit elements of the PB are also awarded more evolutionary flexibility.

In summary, all developmentally defined large-field elements of the PB are identifiable across taxa, albeit with noteworthy species-specific differences. This suggests that the conserved neuroblast lineages producing them represent flexible nodes in the evolution of this neuropil.

#### Large-field elements of the central body upper division

In non-*Drosophilids* the large-field neurons of the FB are collectively referred to as TU neurons (tangential neurons of the CBU). They are classified into eight major groups based on the location of the cell body clusters: TU_VES_, TU_CRE1_, TU_CRE2_, TU_PS1_, TU_PS2_, TU_PS3_, TU_LAL_, and TU_SLP_ (von Hadeln et al. 2020). Such a classification, aided by descriptions of their fibers, enable most of these neurons/groups to be homologized to the *Drosophila* major lineages that form the large-field neurons of the FB (Fig. 8a). Aside from the overall morphology, and neurotransmitters in some instances, little is known about the functional significance of these neurons (el Jundi et al. 2018; von Hadeln et al. 2020; Hensgen et al. 2021a, b).

Somata of the TU_VES_ neurons are located ventro-anteriorly near the vest (VES) of the ventral cerebrum (near the TL1 soma). They extend projections dorsally through the LAL towards the CB via one of the isthmus tracts—characteristic of the BAmv1 lineage. Within this cluster, the TU_VES_1 neuron extends spiny dendritic arbors into the upper LAL (ULAL) and the CRE (wrapping around the medial lobe of the MB), subsequently arborizing in the IIa and IIb layers of the CBU (von Hadeln et al. 2020). This morphology most closely resembles the *Drosophila* FB4H neurons (possibly FB4G and FB4F as well). The dendritic arbors of the TU_VES_2 neurons also innervate the ULAL and the CRE. However, unlike the TU_VES_1 neurons, the ULAL arborization extends more ventrally and the CRE arborization is far sparser. These neurons target the layer III of the CBU and are morphologically reminiscent of the fly FB2D neurons. The TU_VES_3 neurons extend broad arbors into the PS, lateral complex, ventrolateral protocerebrum (VLP), and CRE. Their CBU innervation is predominantly in the III layer. No *Drosophila* BAmv1 FB neuron extends as far ventro-posteriorly as the TU_VES_3 neurons—reflecting a potential species-specific circuit modification. The closest match, owing to its broad ventrally extending arbors in the LAL, is the FB4I neuron type. Finally, the TU_VES_4 neuron traverses the isthmus tract 4, extends spiny arbors into the SIP and very weakly into the CRE, and innervates the layer Ia and the anterior lip (ALI). Such morphology is displayed by the FB6O neurons in the fly. The TU_VES_2 and TU_VES_4 neurons have also been identified in the honeybee brain, arborizing in the ventro-posterior and dorso-anterior regions of the CBU (Hensgen et al. 2021a). Furthermore, the TU-avm cluster, described in the butterfly *Danaus plexippus*, also corresponds to the BAmv1 lineage. The two identified neuron types interconnect the LAL and anterior inferior protocerebrum to layer III of the CBU (Heinze et al. 2013).

An important aim of identifying the homologous neurons across the taxa was to map the CBU and FB layers. Owing to variable tilts in the neuraxis in different species, a one-to-one comparison of the neuropils is complicated. However, using the lineage tracts and the somata descriptions, we repeated the homologizing exercise (presented above for TU_VES_ neurons) for all TU neurons, which broadly corroborates: (1) the structured tiling of CBU input domains to the different layers (as in *Drosophila* FB_ID_, Fig. 5, and also hinted in the flesh fly by Phillips-Portillo and Strausfeld (2012)); and (2) the mapping of the locust CBU layers to fly FB layers across lineages (with few exceptions). Neuron matches are provided in tabular form for easy perusal (Table 3). Given these features, we speculate that the dorso-ventral axis of the FB of the fly maps onto the antero–posterior axis of the locust CBU – with layer III corresponding to the ventral FB, layer IIa/b corresponding to the intermediate FB, and layer Ia/b/ALI corresponding to the dorsal FB. Similar analysis of the collection of butterfly neurons, suggests that the dorso-ventral axis of the CBU is similar to that of the fly FB. The following descriptions of the TU neurons primarily highlight their tract morphology and the rationale of matching them to a corresponding fly lineage.

**Table 3:**
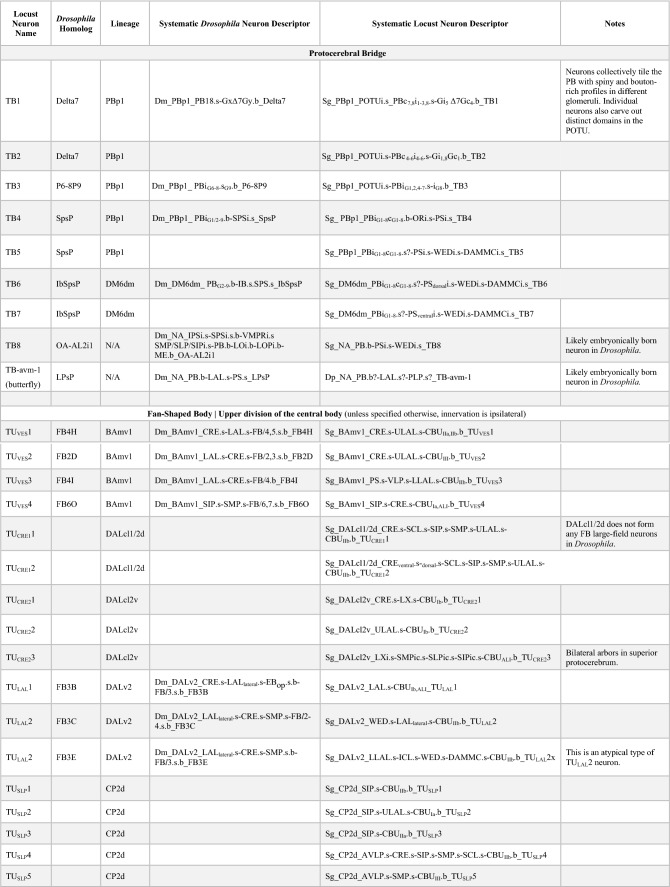

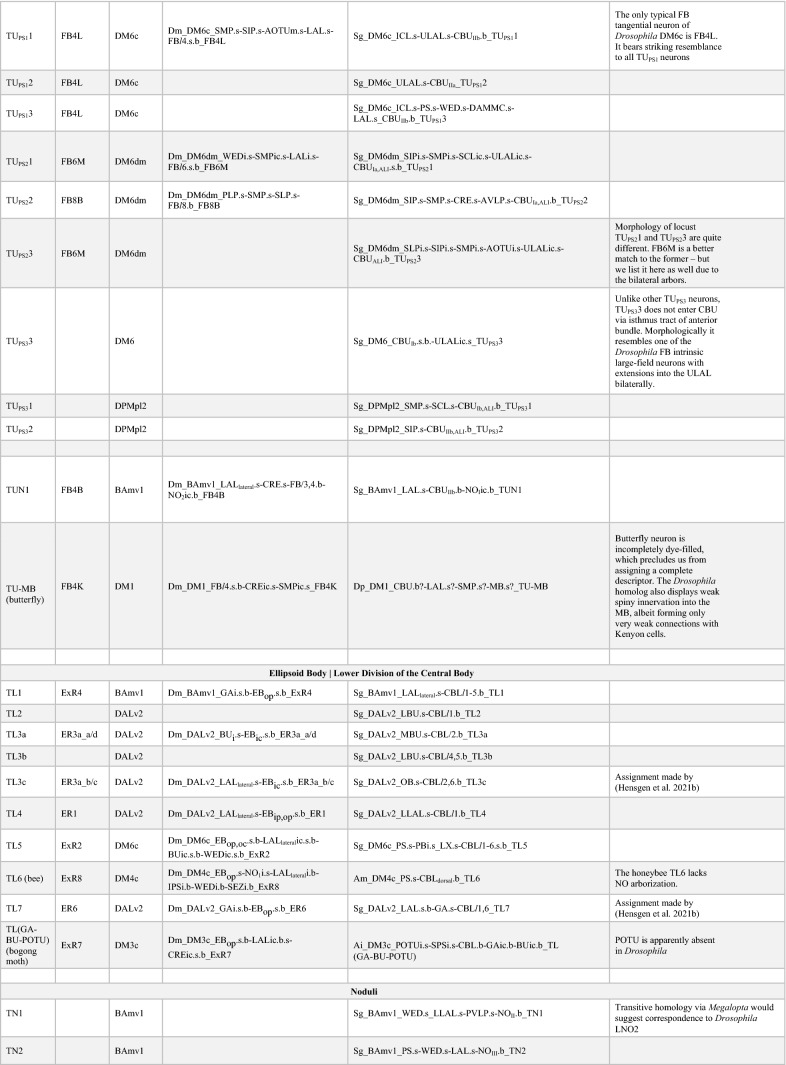
Homologous neurons between *Drosophila* and *Schistocerca* as well as their proposed systematic descriptors

Neurons of the groups TU_CRE1/2_, have somata located laterally of the CRE. The TU_CRE2_ neurons project along the “anterior bundle 3” (von Hadeln et al. 2020), continuing “in front” of the alpha/alpha' lobe (Fig. 8a). They innervate the Ia, Ib, and ALI of the CBU—corresponding to the dorsal FB of the fly. These characteristics suggest that TU_CRE2_ are homologous to the ventral hemilineage of DALcl2 (DALcl2v). TU_CRE1_ neurons extend their tract “behind” the alpha/alpha’ lobe of the MB and continue medially towards the CBU via the isthmus tract (LE) 5 (Fig. 8a). This tract morphology is similar to DALcl1/2d—neither of which generate FB neurons in *Drosophila*.

TU_LAL_ neurons (not to be confused with the TuLAL neurons mentioned above) have somata in the anterior cortex, next to the TL2-4 neurons. They follow the same trajectory as the TL2-4 neurons, and form part of the isthmus tract 2, identifying them as being part of the DALv2 lineage (von Hadeln et al. 2020). The dendritic tufts of these neurons bear morphological similarity to the mechanosensory ER1/TL4 neurons—with one neuron type innervating well into the WED and antennal mechanosensory and motor center (AMMC). A similar soma location, trajectory, and slender LAL and CBU innervation are also displayed by the butterfly TU-aim-(IV) neuron, positing the TU-aim cluster as the DALv2 homolog in Lepidoptera (Heinze et al. 2013).

The TU_PS1_ and TU_PS2_ locust neuron groups, as well as the TU-pvm butterfly cluster, have their somata in the posterior cortex near the PS and project forward, following the same trajectory as the TL5 neurons (see above). This is characteristic of the DM6 lineage, showing shared origins of this set of TU neurons across the three species (Heinze et al. 2013; von Hadeln et al. 2020).

Two additional clusters of locust FB large-field neurons, TU_PS3_ and TU_SLP_, are located laterally of the presumed DM6 lineage. TU_PS3_ somata lie near the postero-medial surface of the calyx while those of the TU_SLP_ flank the calyx laterally (Fig. 8). The former group projects fibers along the superior medial protocerebrum, where they converge with the tract formed by the latter group and diagonally pass through the superior protocerebrum. Tracts then bend medially as the “anterior bundles 1” and “2” towards the FB. The constellation of these two clusters of neurons matches the lineages DPMpl2 (dorsomedial) and CP2d (dorsolateral), respectively. Neuron shapes contained within the two clusters also fall within the projection envelope of these two lineages, with TU_PS3_ neurons emitting dendritic branches towards the SMP and SIP, and TU_SLP_ forming dense tufts in the SIP posteriorly adjacent to the vertical lobe. (Note that TU_PS3_3 is an exception, and is likely a part of DM6. See Table 3 notes for details). A clear instance of the CP2d lineage is represented by the TU-pdl cluster in butterflies (Heinze et al. 2013). Neurons within this cluster have somata dorso-laterally of DM6 (TU-pvm) and extend projections medially towards the CX—splitting into two subpopulations prior to innervating the CBU. Much like the *Drosophila* CP2d dorsal subpopulation, the TU-pvm dorsal subpopulation innervates the dorsal part of the CBU (layers I and II). Interestingly, almost identically to the *Drosophila* CP2d ventral, the TU-pvm ventral subpopulation continues further anterior towards the CBL and turns almost 180° at its ventral surface (owing to the unwrapped CBL and lack of a canal) and proceeds to innervate the ventral layers of the CBU (Heinze et al. 2013). This creates a projection envelope of the TU-pdl cluster, almost identical to the *Drosophila* CP2d clone (Fig. 1d, d'), with a characteristic “gap” in the intermediate layers of the CBU/FB. Although we suspect not, it remains to be seen whether there are other neurons in the butterfly TU-pdl cluster that fill this layer III “gap”.

Not included in these clusters, but an important contributor to the CBU architecture is the locust octopaminergic DUM SA1 neuron (not to be confused with the *Drosophila* AB neuron name)—the likely homolog of the OA-VPM3 *Drosophila* neurons (Homberg et al. 2013). Furthermore, an interesting neuron type in butterflies, TU-MB, has somata located at the medial edge of the PB and extends its tract ventral of the CBU prior to curving dorsally to innervate it from the anterior surface (Heinze et al. 2013). The presumed dendritic tufts of this neuron, despite being incompletely dye-filled, appear to innervate the CRE, dorsal part of the LAL, as well as MB lobes. The tract trajectory suggests that TU-MB is most likely part of the DM1 lineage. Indeed, its morphology bears striking resemblance to the *Drosophila* DM1-FB4K neurons, which even shares very sparse bilateral innervation of the gamma and beta lobes of the MB and weak connections from the respective Kenyon cells. This suggests the presence of direct connections between the MB and the CX, with potential variability across species. Finally, despite not being cataloged in the locust or bee brain, the DAMd2 neurons of the FB/CBU are also evident in the beetle brain (in addition to neurons from BAmv1, DM6 and DALcl2v) (el Jundi et al. 2018).

Through our comparative approach, we were able to identify most developmental units that build the FB across taxa. Indeed, the CBU stands as the most diverse compartment in other species—comprised of at least 8 lineages. As in the other neuropil compartments surveyed here, we note interesting species-specific differences in the morphologies of individual neurons—despite noting deep homology between the tracts and the core elements of the circuit.

#### Neurons of the noduli

In the locust and honeybee, all cataloged large-field neurons that innervate the NO form part of the cluster that we consider homologous to the BAmv1 lineage (von Hadeln et al. 2020; Hensgen et al. 2021a). This includes two types of TN (tangential neurons of the Noduli) neurons (TN1 and TN2) and a single neuron type with shared arborization in the CBU and NO (TUN1). The dendritic tufts of the TN neurons are located in the LAL, PS, and WED, while their axonal arbors target the layers II and III of the NO (von Hadeln et al. 2020). In the sweat bee, *Megalopta genalis*, the TN1 and TN2 neurons have been shown to be responsive to regressive and progressive optic flow stimuli respectively (Stone et al. 2017). We speculate that the former is likely homologous to the *Drosophila* LNO2 neurons, given their functional (Lu et al. 2022) and developmental similarity. Homologs of the DALv2/3pr neurons that innervate the NO in *Drosophila* (Fig. 6), if they exist in other species, are yet to be identified.

The locust TUN1 dendritic tufts are localized in the LAL and extend varicose arbors in the CBU layer IIb as well as bilaterally into layer I of the NO. The *Drosophila* homologs of TUN1 are likely the FB4B neurons which innervate the 4^th^ layer of the FB and the NO_2_ compartment. This homology strengthens our assignment of the locust CBU IIb layer to the intermediate fly FB layers. Furthermore, it allows us to posit that layer I of the upper unit of the locust NO corresponds to the *Drosophila* NO_2_ compartment. An atypical FB-NO neuron type in *Megalopta*, called the FB-NOc, is also part of the BAmv1 lineage (Sayre et al. 2021). Unlike the FB-NO neurons in locusts and flies, that bilaterally innervate the NO, the sweat bee FB-NOc neuron only appears to unilaterally innervate the noduli cap (NOc) in the contralateral hemisphere. What is more interesting, is the fact that this region is not innervated by the TN1/2 (LNO) neurons (Sayre et al. 2021).

Finally, the locust DUM SA1 octopaminergic neurons, like their proposed *Drosophila* homologs (OA-VPM3), also target the NO (Homberg et al. 2013). In the beetle, the DAMd2-derived CBU neuron also bilaterally extends into the NO (el Jundi et al. 2018).

## Discussion

The current work serves to build upon previous anatomical characterization of the central complex (CX) by analyzing the recently generated synaptic resolution connectomic datasets of the fly brain (hemibrain) from a developmental perspective. Using structural features that reflect the developmental profiles of neurons—their cell body locations, fiber trajectories, and neuropil innervation envelopes—we were able to build a lineage-based (developmental) synaptic atlas of the *Drosophila* CX (e.g., as shown in Supp. Figure 3 we can associate individual synapses in the FB with discrete lineages). Furthermore, we were able to extend such lineage analyses to lower-resolution morphological datasets of individual neurons in other insects and examine the differences in circuit architecture. This provides an entry point to study the functional properties of individual neuron classes as well as the network motifs across taxa to understand the developmental and evolutionary logic of circuit assembly/information flow. Here we discuss the organizational principles of the CX and highlight the unique facets of some of the constituent lineages.

### Categorization and nomenclature of neurons

The grid-like structure of the CX is formed by two orthogonally arranged neuronal populations: (1) neurons spanning the medio-lateral axis and having large splayed-out arbors (Fig. 1a); and (2) neurons with small arbors collectively tiling each compartment and interconnecting the CX along the antero-posterior axis (Fig. 1b). Due to their distinctive CX arbor morphology, these populations are called large- and small-field neurons respectively (Hanesch et al. 1989). Large-field neurons have also been referred to as “tangential” neurons of the CX (Stone et al. 2017; el Jundi et al. 2018; von Hadeln et al. 2020; Hensgen et al. 2021a, b; Sayre et al. 2021; Hulse et al. 2021). Although these two terms are often used interchangeably, here we propose a distinction between them, whereby the term “large-field neuron” is used to describe any neuron with broad splaying arbors in the CX, and “tangential neuron” is reserved for neurons (subset) that also have dendritic tufts located in lateral neuropils outside the CX. The term “intrinsic” would encompass large-field neurons with projection envelopes that remain entirely restricted within the CX volume (Fig. 1a; PBp1 and DM6dm *Drosophila* neurons). Interestingly, the transition between these two categories (“tangential” ↔ “intrinsic”) seems to be a part of the evolutionary repertoire of lineages—as evidenced by the Delta7 and TB1/2 homology across species (see comparative section on protocerebral bridge (PB)).

Some neurons display morphological features that preclude easy classification into small- or large-field populations. For instance, given the lack of columnar organization of the noduli (NO) one cannot really distinguish between small-field and large-field neurons based on NO innervation alone: all neurons branch throughout the entire width of a given compartment. Additionally, in the PB, the *Drosophila* IbSpsP (proposed TB6/7 homologs in other species) neurons are quite atypical. Although they provide inputs to the PB from lateral neuropils, they innervate only a few adjacently located glomeruli. Given the large input domains of these neurons outside the NO and PB respectively, we still consider them “tangential large-field” neurons. Interestingly, in locusts, the PB span of the TB6/7 neurons is wider than that of *Drosophila* IbSpsP neurons. If they are indeed homlogous, this might represent a transition from a typical large-field to small-field innervation pattern—another potential mechanism to generate diversity across taxa.

Beyond this broad categorization, historic as well as recently increased interest in generating high-resolution datasets of neurons in the insect CX presents a challenge in standardizing the nomenclature (identifiers or descriptors) of individual neurons such that they are easily comparable across the species. In previous studies, large-field neurons in species other than *Drosophila* have been given 2 or 3-lettered/numbered names that only indicate their neuropil of innervation (e.g., TB1, TN1, TL1), and only rarely (in the case of the CBU) any information beyond that. In *Drosophila*, Wolff et al. (2015) proposed a standardized neuron nomenclature method that incorporates the innervation domains and synaptic profiles across neuropils. This method removes ambiguity and errors that occur in the literature from assigning common monikers to neurons (see, for example, Omoto et al. (2018) for a historical account of ER-neuron identification and classification). However, it has two limitations that need to be addressed before widespread incorporation into other species. First, as evidenced most prominently in the FB, neurons that share the same projection envelopes often arise from different lineages—indicating different developmental and possibly functional profiles (Fig. 5). In the EB, similar confusion would arise for the ER6 (DALv2) and ExR4 (BAmv1) using the Wolff et al. (2015) nomenclature—given their extremely similar innervation patterns in the GA and EBop. Second, dye-filled labeling of neurons in other species often misses fine processes—underestimating their projection envelopes and leading to incomplete names. For example, the dye-filled labeling of the TB-avm-1 (as well as TU-MB) neuron in *Danaus plexippus*, described by Heinze et al. (2013), only visualizes the large backbone of the neuron. An additional concern arises from the three-lettered and numbered identifiers provided by Scheffer et al. (2020) to unstudied (newly identified) neurons in the hemibrain. For example, PS017 shares a high degree of morphological similarity as well as identical developmental origin (DALd lineage) as the anterior descending neuron cluster (DNa), particularly DNa01/04 (Namiki et al. 2018). However, PS017 is one of 311 unique neuron types, originating from ~ 35 different lineages, and sharing innervation in several other neuropil compartments, named as PSxxx. This nomenclature system, which extends to most neuropil compartments (Scheffer et al. 2020), does not disambiguate between the drastically different groups of neurons, the developmental histories of which may reflect organization into different circuit modules (as we see in the CX input networks). We propose to address these limitations by appending lineage/tract information into the Wolff et al. (2015) and Scheffer et al. (2020) nomenclature systems (while retaining their existing monikers)—to create long-form, unambiguous descriptors of neurons. Given the deep homology between the lineages and tracts across species, this addition will not only enable the community to disambiguate neurons with similar projection envelopes within species but also ease the efforts to identify homologous neurons across taxa (e.g., TL1 neurons in locust cannot be homologous to fly ER-neurons due to different neuroblasts of origin). A sample comparison across species would look like:

Species_lineage_innervation_existing-moniker

Fly: Dm_BAmv1_GAi.s.b-EB_op_s.b_ExR4

Locust: Sg_BAmv1_LAL_lateral_.s-CBL*l*1-5.b_TL1

Where possible we try to implement these lineage-based descriptor assignments in flies and locusts (Table 3). The generation of higher-resolution datasets will require such assignments at scale.

We acknowledge that such lengthy descriptors might not be entirely accessible to the community, and thus choose to retain the moniker for common use (as we have throughout the text). We expect the long-form name to be reserved for introducing neurons or identifiers in databases such as neuPrint+ (Plaza et al. 2022) or insect brain database (Heinze et al. 2021).

### The unique developmental profile of the PB: PBp1 and DM6

We identified two lineages in *Drosophila* (hemibrain) that give rise to PB large-field neurons: PBp1 and DM6. Interestingly, our MARCM efforts to generate a developmental time-course map of the adult brain did not reveal the PBp1 lineage (Lovick et al. 2013; Wong et al. 2013). It has, however, been documented in the MARCM collection generated by Yu et al. (2013) and Ito et al. (2013)—who treat it as a “clonal unit” that builds the adult brain. More importantly than the projection envelopes, our approach in this project (and all previous work) uses characteristic axon tracts, which remain discernable throughout development—despite the absence of certain neuropil compartments such as the PB in the larval brain (Pereanu and Hartenstein 2006; Cardona et al. 2009; Lovick et al. 2013; Hartenstein et al. 2015). In contrast to all other lineages we have described here and previously, no tract has been identified in the larval brain (in the first instar larval connectome dataset or immunohistochemical labeling) that could correspond to PBp1. Hence, it is possible that PBp1 is not an independent neuroblast lineage, but, instead, an intermediate neural progenitor (INP) derivative of one of the DM lineages. PBp1 neurons display pseudo-columnar morphology and synaptic profiles in the PB, which would be akin to the true columnar neurons generated by another DM1-4 INP (forming the DM1-4c tracts). In other species, the location of the somata of the TB1-4 neurons is shifted more dorso-laterally of the PB than one would expect based on the neuraxis tilt. This slight variability in an otherwise very strict tract mapping across species further suggests atypical origins.

PBp1 neurons also display “tangential” ↔ “intrinsic” morphologies across taxa correlated to their navigational strategies. The external arbors of the PBp1 tangential neurons, TB1/2 in locusts, butterflies, ants, and bees occupy the posterior optic tubercle (POTU). This compartment is apparently missing in *Drosophila*—reflecting the role of PBp1 and potentially other lineages in shaping neuropil compartments and their computations across the brain. The variability of these neurons across taxa further suggests that PBp1, a likely consequence of their INP developmental profile, are flexible nodes in the evolutionary architecture of the CX and accessory compartments.

Like the PBp1 neurons, the IbSpsP neurons of the PB are also not detectable in any of the MARCM clones in our or Yu et al. (2013) and Ito et al. (2013) collections. Our tract and neuropil entry points analysis in the EM volume places these neurons as part of DM6 (dorso-medial), another type II lineage. These observations suggest that the IbSpsP neurons are also likely derivatives of one of the INPs generated by the DM6 neuroblast. In *Drosophila*, their somata are located just adjacent to the midline, ventral of the PB. In locust, the somata of the homologous TB6/7 neurons are located further laterally, close to other DM6 tracts (DM6c and DM6dl). The variability in the span of their PB arbors across species illustrates the evolutionary flexibility of these INP derivatives of DM6.

Neuromodulatory elements of the PB, which in *Drosophila* we suspect are embryonically born, are also conserved across species. The larval brain does not have a PB (although a primordium of this structure is detectable using N-cad antibody; Andrade et al. (2019)). Thus, this incorporation represents a major transdifferentiation event during pupal development. Overall, it appears as though all the elements that constitute the PB network across taxa are quite atypical in their developmental profiles and highly dedicated—while retaining immense flexibility—to modulating and reformatting the vector computations occurring in this neuropil.

### BAmv1: sequential assembly of the CX

The BAmv1 lineage is not only spatially very distinct across taxa, but the constituent neurons also display a low degree of variability. BAmv1 is also among the broadest lineages of the CX, despite being type I—not producing INPs that have their own distinct fates, which typically results in a large increase in lineage size. Spanning almost all CX compartments, the computations performed by BAmv1 neurons may be less tolerant to variability—putatively making it easier to bridge functional data across taxa.

Recent examination of the temporal profile of the BAmv1 neuroblast in *Drosophila* reveals the sequential assembly patterns of the CX (Lee et al. 2020). Among the first BAmv1 neurons to be born are the FB-NO neurons, which arborize in the intermediate layers of the FB and extend arbors into the NO. Subsequently, this lineage predominantly forms neurons that target the dorsal layers of the FB, before proceeding to make the neurons innervating the intermediate, and finally the ventral layers. While no detailed birth-order series exists for any other lineage targeting the FB, we speculate that they too, would follow a similar trend. The formation of the FB-NO neurons first shows the tight coupling of these two neuropils. Early formation of the intermediate layers also suggests an increased significance structurally, and possibly functionally, of inputs directly from the MBONs. These connections likely mediate navigational responses tied to environmental/chemical salience cues—a critical computation for nutrition and survival. The last neurons to be formed by BAmv1 are the neurons of the AB, highlighting an additional role/molecular mechanism in this lineage dedicated to generating brain asymmetries.

The invariability of BAmv1 thus highlights the structural and functional foundation that lineages can establish across evolutionary timescale.

### Developmental organization of the columnar neurons of the CX

Although not the focus of this study, the orthogonally arranged small-field neurons of the CX have garnered significant attention. Corroborating previous studies, we were also able to assign them to four dorsomedial lineages, DM1-4 (central), on either side of the brain. Unlike most other lineages, DM1-4 all give rise to (almost) all of the small-field neuron types. These populations of complementary small-field neurons from each DM lineage subdivide the CX into quadrants (Ito et al. 2013; Yu et al. 2013; Yang et al. 2013; Wong et al. 2013; Andrade et al. 2019). Of particular interest within these populations are the tiling offsets, called “phase-shifts”, that they generate among each other and across compartments (Pisokas et al. 2020; Sayre et al. 2021; Hulse et al. 2021). It is these phase-shifts that are responsible for the vector computations that underlie the representation of various forms of spatial information (e.g., heading direction and traveling direction) (Seelig and Jayaraman 2015; Turner-Evans et al. 2017, 2020; Green et al. 2017; Fisher et al. 2019; Kim et al. 2019; Lu et al. 2022; Lyu et al. 2022). How are these unique phase-shifts between 68 different small-field (and pontine) neuron populations assembled? The quadrant-restricted projection envelopes of DM1-4 already suggest potential lineage-based mechanisms to achieve this without genetically hard-coding combinatorial IDs for each synaptic interaction—features that are essential to maintaining the ability to flexibly form varying circuits across taxa. We are examining these principles using a similar approach and soon hope to present the results in a follow-up paper.

### Mechanisms for generating diversity

The sequential gene expression profiles of each neuroblast specify the properties of the neurons born during specific temporal windows (Broadus and Doe 1995). Modifications to the division number with a given transcriptional profile can allow the expansion or compression of certain populations of neurons (sublineages) (Truman and Ball 1998). Concomitant changes to the genes being expressed can enable the formation of different or even novel neuron types/morphologies (Sullivan et al. 2019). Modifications to circuit architecture can also arise by hemilineage apoptosis or degeneration mechanisms (Kumar et al. 2009a, b). More broad-scale diversification, such as the rapid expansion of the mushroom bodies (MB) in some species could arise from the transition of corresponding neuroblasts from type I to type II division fates. Indeed, the MB proximity in other species are lined by ~ 500 dividing units, likely representing the INP—that could arise from the four MB neuroblasts (Farris 2013; Ito et al. 2013; Yu et al. 2013; Wong et al. 2013; Lovick et al. 2017; Farnworth et al. 2022).

The ease of modifications to any of these parameters/properties enables insect brains to undergo rapid evolution—and enable the incorporation/deprecation of novel ecological niches, sensory modalities, appendages, and behavioral profiles.

### The lineage-mechanism as a central concept towards understanding the development and evolution of the insect brain

Lineages form the building blocks of insect (and potentially pancrustacean) nervous systems. Neurons of a typical (type I) lineage form one or two tracts (depending on whether or not both hemilineages are maintained); type II lineages where INPs are inserted in between the neuroblast and its progeny have more tracts. Tracts of most lineages can be homologized between different species (Farnworth et al. 2022), and thereby serve as the “anchor-points” of the corresponding lineages even in the absence of sophisticated genetic tools used to individually mark these lineages. The current work has focused on the central complex as a domain where the lineage-based assembly of tracts and neuropil compartments can be applied to great advantage towards gaining a deeper understanding of brain circuitry, as well as recognizing homologies across insect species.

Lineages tile the overall volume of the brain—most neurons of a given lineage are focused on a few neuropil compartments. This focus can be very pronounced for some lineages/hemilineages (e.g., the MB lineages, or the lineages generating projection neurons connecting the antennal lobes, calyx, and lateral horn), or less so in others. However, most lineages focus the majority of their neurons on a discrete neuropil volume. In the context of the CX input neurons analyzed here, this principle applies strictly to the two hemilineages, DALcl1d and DALcl2d, which generate virtually all input to the EB large-field lineage DALv2. Applying the same idea to the large-field neurons of the FB, which are more numerous and, correspondingly, have a dendritic input domain (FB_ID_) much larger than the BU—it is no wonder that over 30 lineages constitute their synaptic input profile. That being said, input to large-field neurons of the other CX compartments is fairly restricted in terms of number of lineages supplying this input.

One can detect that, although a small number of homologous lineages produce the neurons forming input pathways and intrinsic processing units of the CX in different insects, neurons of these lineages display differences which may reflect the ecological niche of the animal. We hypothesize that selective pressures can drive genetic changes resulting in the modification of neuroblast patterning in a way that varies the number of cells of a given type or modifications in wiring, representing “tuneable-knobs” in the genotype–phenotype map of the brain.

## Supplementary Information

Below is the link to the electronic supplementary material.

**Supp. Fig. 1** 1Lineages that contain the majority of CX large-field neurons. **a-c** DALv2. **d-f** DALcl2. **g-i** BAmv1. **j-l** CP2. **m-o** DALcm1. **p-r** BAmd1. **s-u** DPMpl2. **v-x** DM6. Left column presents z-projections of frontal confocal sections of Drosophila brain at level of fan-shaped body. Shown are GFP-labeled MARCM clones of the lineages indicated at bottom left. Neuronal cell bodies are rendered in magenta, fiber tracts and arborizations in green. Panels of middle and right column present digital/in-silico clones of the same lineages as those shown in left column; all parts (cell bodies, tracts, terminal branches) are superimposed in middle column (green rendering), whereas tracts only are shown on the right (white rendering). For abbreviations see Table 1 (TIF 57367 KB)

**Supp. Fig. 1 continued** (TIF 55572 KB)

**Supp. Fig. 2** Strength of ER neuronal output on E-PG (“compass”) neurons. Note consistently stronger output of ER classes that receive input from DALcl1d TuBu neurons (magenta). Two exceptions are presented by DALcl2d-innervated ER3p (high output to E-PG) and DALcl1d-innervated ER5 (low output to E-PG) (TIF 15249 KB)

**Supp. Fig. 3** Distribution of output synapses of the fan-shaped body large-field neurons across the different layers. **a** All lineages grouped. Isolated by individual neurons of: **b** BAmv1 lineage; c CP2d lineage. In all panels FB layers are shown along the vertical axis; lineages (in **a**) or individual neurons (in **b** and **c**) are shown on the horizontal axis (TIF 18975 KB)

**Supp. Fig. 4** DALv2 neurons of the FB. Linage DALv2 generates neuron classes with likely mechanosensory-driven dendritic input in the lateral LAL and output to the EB (ER1, ER3a) and to the FB (FB3B, FB3C, FB3E) (TIFF 149132 KB)

**Supp. Fig. 5** Topography of columnar neurons of the Asymmetrical Body (AB). Columnar neurons FS4A and FS4B (top two rows) and vDeltaA_a and vDeltaA_b neurons (bottom two rows) (TIFF 108058 KB)

**Supp. File 1** 3D interactive rendering of TuBu neurons, color-coded by lineage (DALcl1d : magenta and DALcl2d : blue) (HTML 11327 KB)

**Supp. File 2** 3D interactive rendering of DALv2 central complex innervating neurons.(HTML 29250 KB)

**Supp. File 3** 3D interactive rendering of BAmv1 central complex innervating neurons.(HTML 35518 KB)

**Supp. File 4** 3D interactive rendering of DM6dm central complex innervating neurons.(HTML 24680 KB)

**Supp. File 5** 3D interactive rendering of DM6c central complex innervating neurons.(HTML 16473 KB)

**Supp. File 6** 3D interactive rendering of DM6dl central complex innervating neurons.(HTML 10660 KB)

**Supp. File 7** 3D interactive rendering of DM4c central complex (large-field) innervating neurons. (HTML 10395 KB)

**Supp. File 8** 3D interactive rendering of DM4d central complex (large-field) innervating neurons.(HTML 10573 KB)

**Supp. File 9** 3D interactive rendering of DM3c central complex (large-field) innervating neurons. (HTML 10647 KB)

**Supp. File 10** 3D interactive rendering of PBp1 central complex innervating neurons.(HTML 16251 KB)

**Supp. File 11** 3D interactive rendering of DALcl2v central complex innervating neurons.(HTML 27655 KB)

**Supp. File 12** 3D interactive rendering of CP2d central complex innervating neurons.(HTML 26956 KB)

**Supp. File 13** 3D interactive rendering of DPMpl2 central complex innervating neurons.(HTML 15616 KB)

**Supp. File 14** 3D interactive rendering of DALv2pr central complex innervating neurons.(HTML 10218 KB)

**Supp. File 15** 3D interactive rendering of DALv3pr central complex innervating neurons.HTML 9777 KB)

**Supp. File 16** 3D interactive rendering of DAMd2 central complex innervating neurons.(HTML 10672 KB)

**Supp. File 17** 3D interactive rendering of DALcm1 central complex innervating neurons.(HTML 9446 KB)

**Supp. File 18** 3D interactive rendering of BAmd1 central complex innervating neurons.(HTML 9047 KB)

**Supp. File 19** 3D interactive rendering of DM1 central complex (large-field) innervating neurons.(HTML 9503 KB)

**Supp. Table 1** All hemibrain CX large-field neurons with their lineage assignments. Neurons were included in our list if they: (1) innervated any of the CX compartments, (2) formed more than a total of 15 T-bars and post-synaptic densities in said compartments, and (3) formed at least one output connection, to a single neuron, with a strength greater than 5 synapses.(CSV 53 KB)

**Supp. Table 2** Hemibrain TuBu neurons with their lineage assignments. (CSV 4 KB)

**Supp. Table 3**Assignment to dorsal and ventral subpopulations of DALcl2v fan-shaped body neurons.  (XLSX 12 KB)

**Supp. Table 4** Assignment to dorsal and ventral subpopulations of CP2d fan-shaped body neurons. (XLSX 12 KB)
