# Supplementary figures and images for: Lineages to circuits: the developmental and evolutionary architecture of information channels into the central complex
Source: J Comp Physiol A Neuroethol Sens Neural Behav Physiol. 2023 Mar 17;209(4):679–720. doi: 10.1007/s00359-023-01616-y (PMC10354165; doi:10.1007/s00359-023-01616-y)

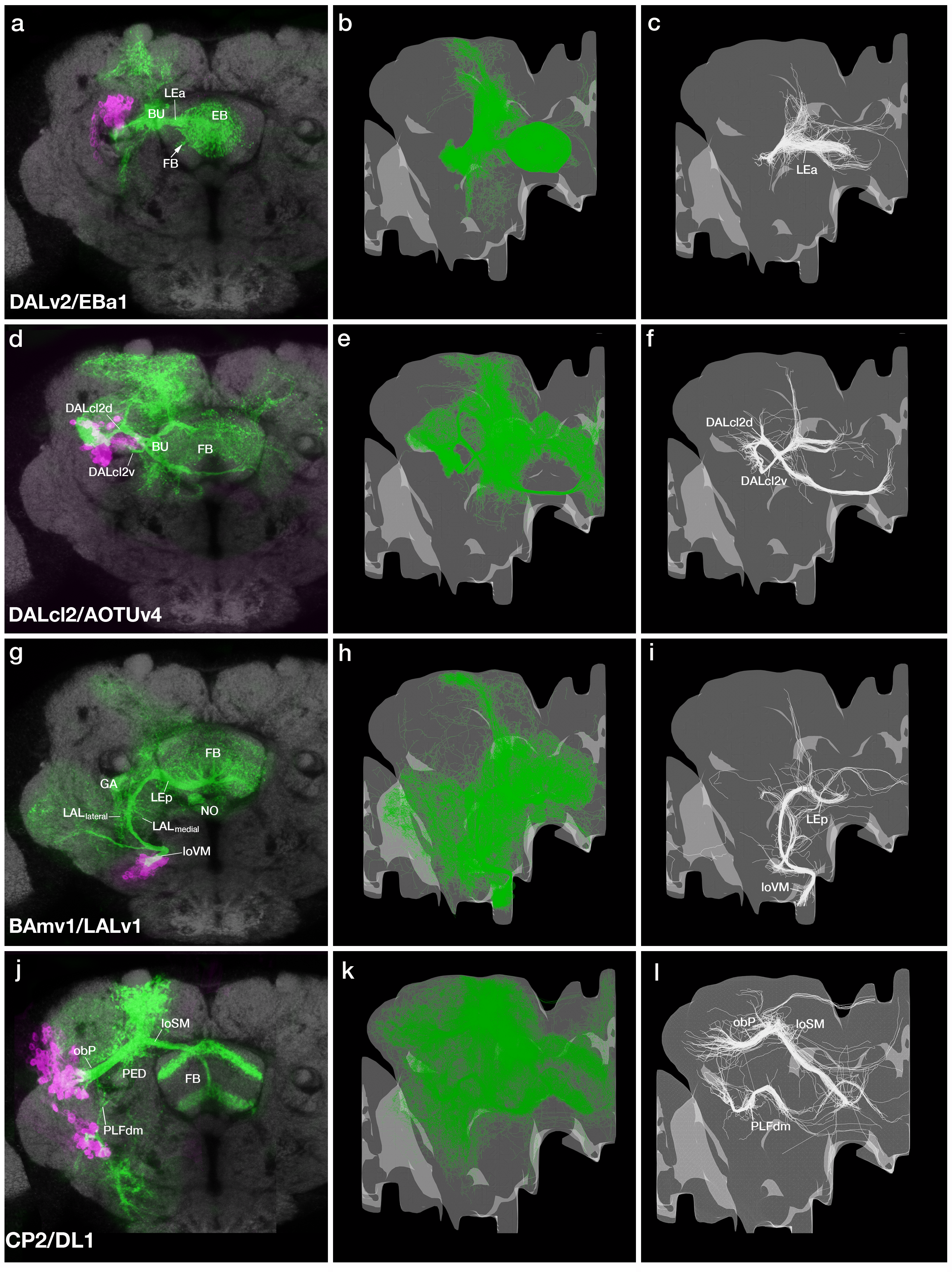

Supplement: Supplementary file 1 — Supp. Fig. 1 1Lineages that contain the majority of CX large-field neurons. a-c DALv2. d-f DALcl2. g-i BAmv1. j-l CP2. m-o DALcm1. p-r BAmd1. s-u DPMpl2. v-x DM6. Left column presents z-projections of frontal confocal sections of Drosophila brain at level of fan-shaped body. Shown are GFP-labeled MARCM clones of the lineages indicated at bottom left. Neuronal cell bodies are rendered in magenta, fiber tracts and arborizations in green. Panels of middle and right column present digital/in-silico clones of the same lineages as those shown in left column; all parts (cell bodies, tracts, terminal branches) are superimposed in middle column (green rendering), whereas tracts only are shown on the right (white rendering). For abbreviations see Table 1 (TIF 57367 KB) [file 359_2023_1616_MOESM1_ESM.tif]

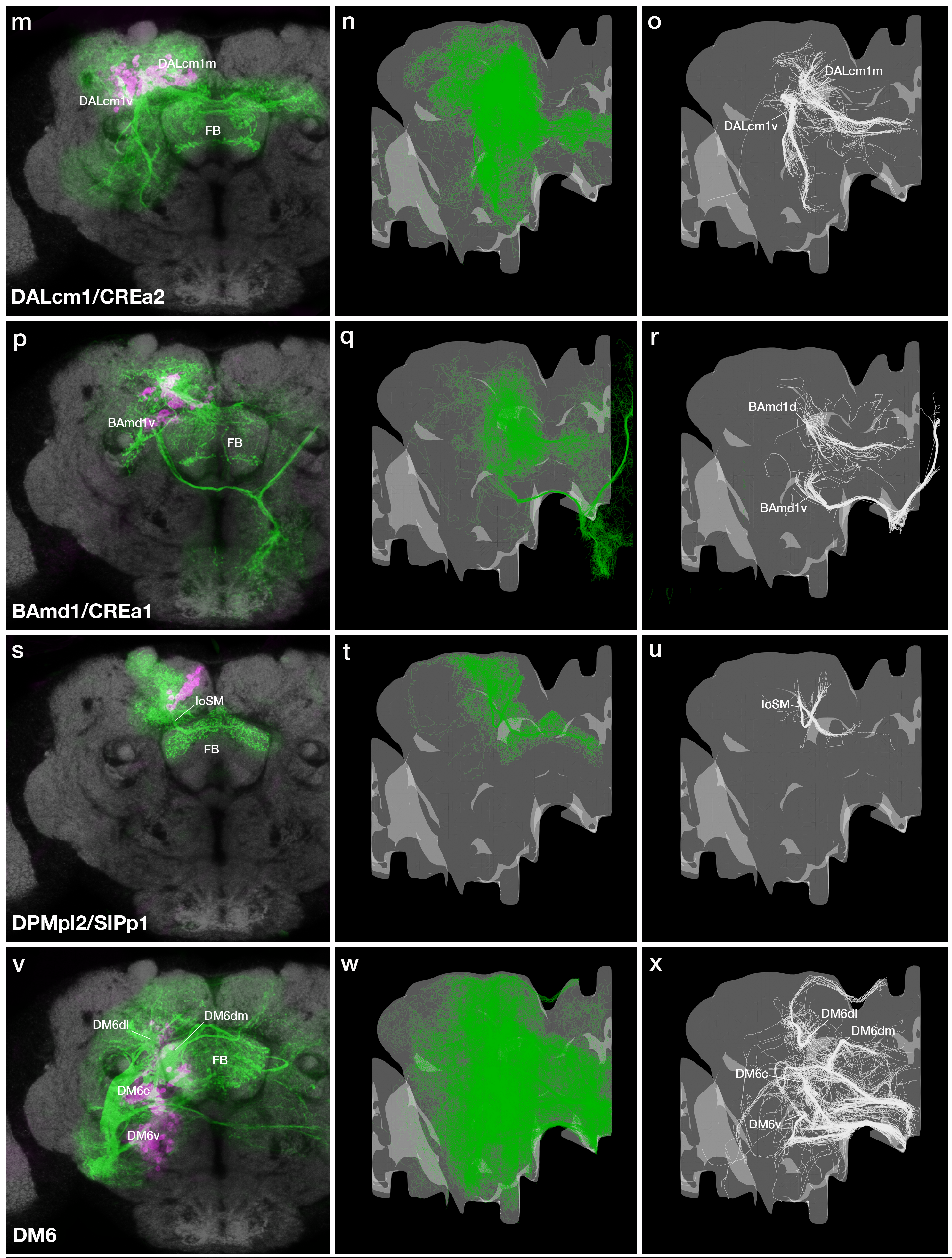

Supplement: Supplementary file 2 — Supp. Fig. 1 continued (TIF 55572 KB) [file 359_2023_1616_MOESM2_ESM.tif]

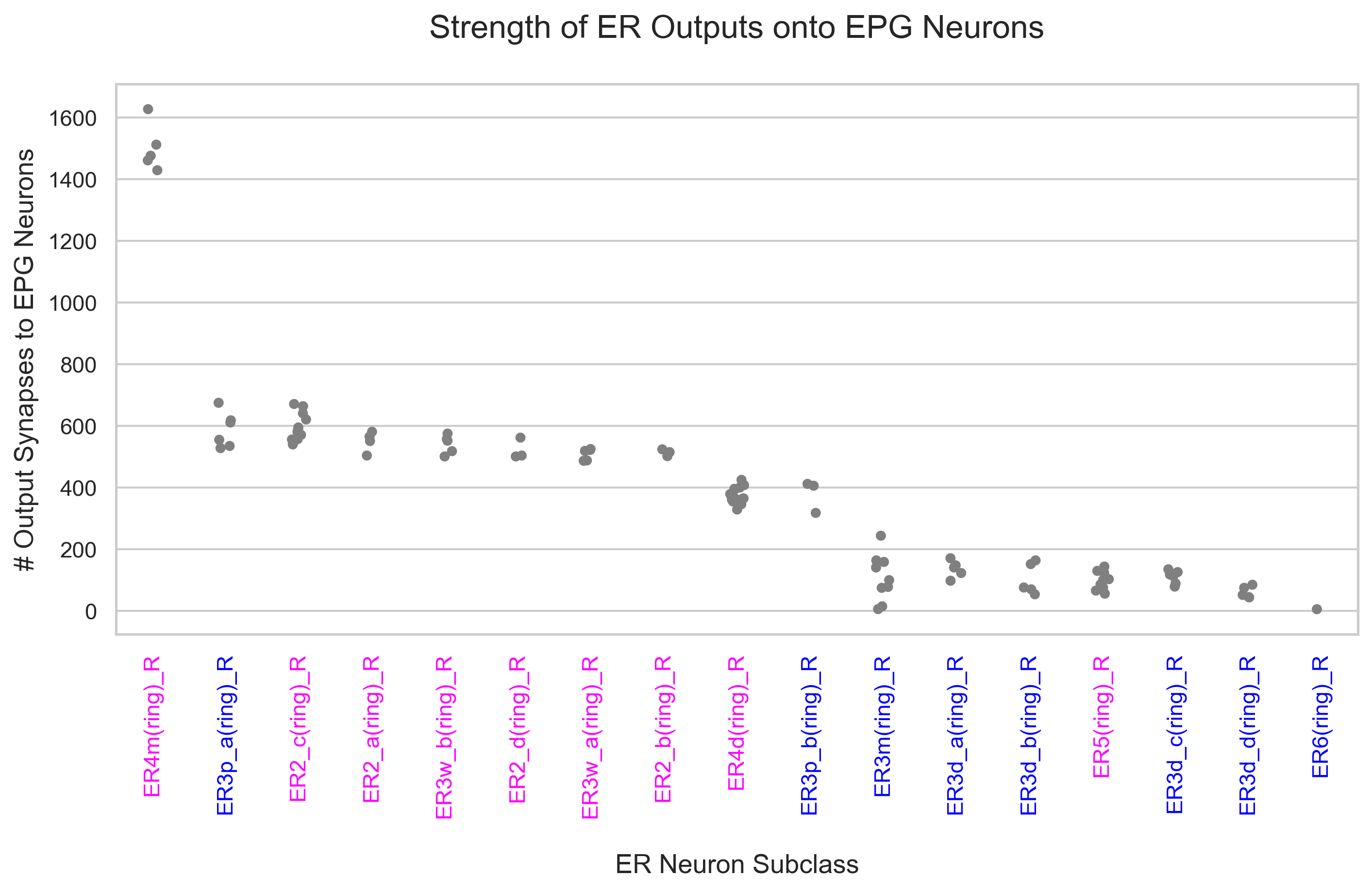

Supplement: Supplementary file 3 — Supp. Fig. 2 Strength of ER neuronal output on E-PG (“compass”) neurons. Note consistently stronger output of ER classes that receive input from DALcl1d TuBu neurons (magenta). Two exceptions are presented by DALcl2d-innervated ER3p (high output to E-PG) and DALcl1d-innervated ER5 (low output to E-PG) (TIF 15249 KB) [file 359_2023_1616_MOESM3_ESM.tif]

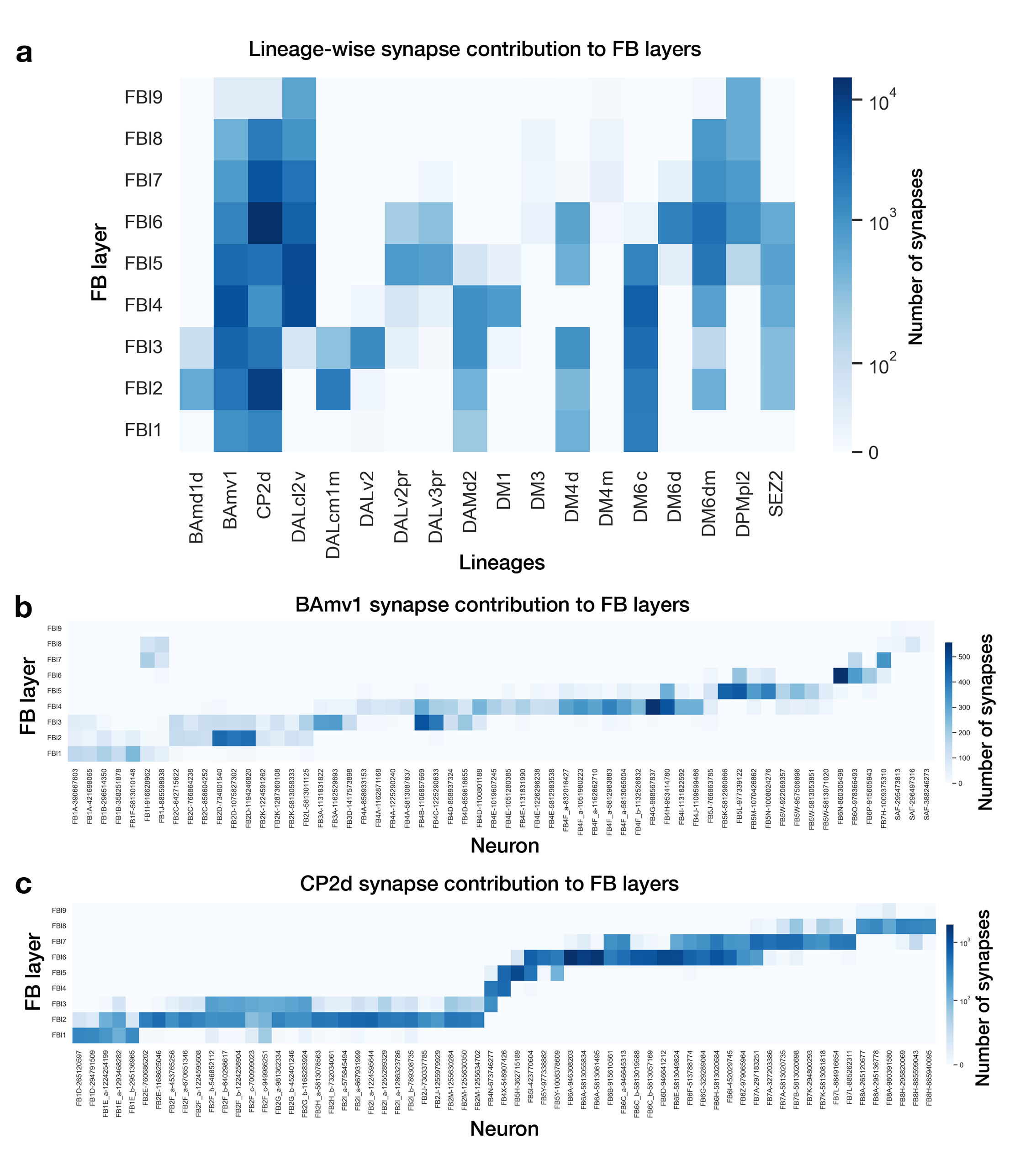

Supplement: Supplementary file 4 — Supp. Fig. 3 Distribution of output synapses of the fan-shaped body large-field neurons across the different layers. a All lineages grouped. Isolated by individual neurons of: b BAmv1 lineage; c CP2d lineage. In all panels FB layers are shown along the vertical axis; lineages (in a) or individual neurons (in b and c) are shown on the horizontal axis (TIF 18975 KB) [file 359_2023_1616_MOESM4_ESM.tif]
